# Single-Nucleus Transcriptomics Reveals Granulosa Cell Heterogeneity and Microenvironmental Remodeling Across Bovine Ovarian States

**DOI:** 10.3390/biology15151327

**Published:** 2026-08-06

**Authors:** Yanchun Bao, Fengying Ma, Xiaoxia Qi, Mingjuan Gu, Lin Zhu, Caixia Shi, Risu Na, Wenguang Zhang

**Affiliations:** 1 College of Animal Science, Inner Mongolia Agricultural University, Hohhot 010018, China; byc107054@163.com (Y.B.);; 2 Inner Mongolia Engineering Research Center of Genomic Big Data for Agriculture, Inner Mongolia Agricultural University, Hohhot 010018, China; 3 Inner Mongolia Saikexing Livestock Breeding and Reproductive Biotechnology Research Institute Co., Ltd., Hohhot 011517, China; 4 National Center of Technology Innovation for Dairy, Hohhot 010100, China

**Keywords:** Bos taurus, single-nucleus RNA sequencing, ovary, granulosa cells, cell-cell communication, metabolic reprogramming

## Abstract

Ovarian function is essential for fertility in dairy cattle, yet the cellular and molecular features associated with physiological ovarian states and ovarian dysfunction remain incompletely characterized. In this study, serum and follicular-fluid hormone measurements showed distinct endocrine profiles among ovarian states, and the follicular-fluid estrogen to progesterone was highest during the follicular phase and lowest in luteal and luteal cystic ovaries. Then, single-nucleus RNA sequencing was performed on ovarian tissues from 18 Holstein cows representing follicular, luteal, mid-gestation pregnancy, inactive, and luteal cystic states. After quality control, 154,054 nuclei were retained for cell-type annotation, granulosa cell (GC) subclustering, trajectory inference, co-expression analysis, ligand-receptor and ligand-target prediction, and transcriptome-based metabolic flux estimation. Twenty-seven ovarian cell clusters and five GC subtypes were identified, with state-associated variation in relative nuclear composition and transcriptional profiles. Luteal cystic ovaries showed a higher relative representation of immune cells and enrichment of inflammation-related transcriptional signatures, whereas inactive ovaries exhibited lower levels of predicted intercellular communication. GC analyses indicated differences among ovarian states in transcriptional programs related to proliferation, steroidogenesis, extracellular-matrix organization, inflammation, and metabolism. Transcriptome-based metabolic inference further suggested subtype-associated variation in tricarboxylic acid cycle, lipid, polyamine, phosphoinositide, and gamma-aminobutyric acid-related pathways. This study provides a multi-state single-nucleus transcriptomic resource for bovine ovarian research and identifies candidate cell populations and molecular features for future experimental validation.

## Simple Summary

Dairy cow fertility depends strongly on ovarian function, but the ovary contains many different cell types that change with the reproductive state and disease. This study examined ovaries from Holstein cows in follicular, luteal, pregnancy, inactive, and luteal cystic states using single-nucleus RNA sequencing, a method that measures gene activity from thousands of individual cell nuclei. We built a multi-state cell map of the bovine ovary and found that granulosa cells, which support follicle growth and hormone production, differed markedly among ovarian states. Inactive ovaries showed weaker predicted communication among ovarian cells, whereas luteal cystic ovaries showed stronger signs of immune and inflammatory remodeling. The study provides a cellular resource for understanding how normal ovarian function and ovarian disorders differ in dairy cattle, and it highlights candidate cell populations and pathways that may be tested in future reproductive biology and animal breeding research.

## 1. Introduction

In high-producing Holstein cows, decades of genetic selection for milk yield have been associated with declined reproductive performance, characterized by prolonged calving intervals and increased culling due to reproductive failure [1,2,3]. Among the reproductive disorders affecting postpartum dairy cattle, ovarian dysfunction—particularly inactive ovaries and cystic ovarian disease—represents a major cause of subfertility. Inactive ovaries reflect failure to resume follicular waves after calving, whereas cystic structures arise from persistent follicular or luteal tissues that do not regress or ovulate [4,5]. These conditions are associated with disrupted hypothalamic-pituitary-ovarian signaling and impaired intra-ovarian communication [6].

Granulosa cells (GCs) are the principal somatic cell population supporting follicle growth and oocyte competence. As the primary steroidogenic cells in growing follicles, GCs produce estradiol (E_2_) in response to follicle-stimulating hormone (FSH) and mediate pathways controlling folliculogenesis and ovulation [7,8,9]. Cross-species single-cell studies have suggested that GCs are transcriptionally dynamic and functionally diverse. In goats, single-cell atlases revealed GC-specific enrichment of steroidogenic and apoptotic pathways [10]. In cattle, single-nucleus RNA sequencing (snRNA-seq) has shown that GC transcriptional programs differ markedly between cyclic and pregnant states [11]. In bovine cystic follicles, GCs exhibit altered steroidogenic gene expression, dysregulated apoptosis, and aberrant inflammatory responses, contributing to follicular persistence [12]. Recent studies have further resolved GC populations into subtypes including cumulus, mural, atretic, and luteinizing GCs [13]. These previous studies have provided important insights into specific follicular compartments, GC populations, reproductive stages, or ovarian disorders. However, most have focused on selected cell types, individual follicular stages, comparisons between a limited number of physiological conditions, or isolated cystic follicles. Consequently, the cellular and transcriptional differences among normal cyclicity, pregnancy, inactive ovaries, and luteal cystic ovaries have not been extensively compared within a unified whole-ovary single-nucleus framework. In particular, it remains unclear how GC subtype composition, transcriptional programs, and the surrounding ovarian microenvironment differ across these physiological and pathological conditions.

Traditional histological and bulk transcriptomic approaches provide averaged signals and may therefore obscure rare cell populations and transitional transcriptional states [14]. Although single-cell RNA sequencing (scRNA-seq) has advanced reproductive biology [15,16], its application to structurally complex bovine ovarian tissue is limited by extensive extracellular matrix and dissociation-related cell loss. SnRNA-seq provides a practical alternative for profiling frozen ovarian tissues and may reduce biases associated with whole-cell dissociation [14].

Accordingly, this study aimed to construct a single-nucleus transcriptomic atlas of Holstein cow ovaries across follicular, luteal, pregnancy, inactive, and luteal cystic states, characterize major ovarian cell populations and GC subtypes, and identify state-associated differences in cellular composition, transcriptional programs, intercellular signaling, and predicted metabolic features. These analyses were intended to generate biologically relevant hypotheses rather than establish direct causal mechanisms. The resulting atlas provides a cellular reference for future validation studies and may support a better understanding of ovarian dysfunction and fertility-related traits in dairy cattle.

## 2. Materials and Methods

### 2.1. Ethics Statement

All procedures involving animals were conducted in strict accordance with institutional guidelines approved by the Animal Welfare and Ethics Committee of Inner Mongolia Agricultural University (Approval No. NND2025254).

### 2.2. Animal Management

A total of 49 multiparous Holstein dairy cows, with an average age of approximately 5 years, were initially screened at Xuyi Animal Husbandry Co., Ltd. (Bayannur, Inner Mongolia, China) between September and October 2025. Ovarian status was evaluated using transrectal ultrasonography with a Mindray DP-50Vet ultrasonic diagnostic system (Mindray Animal Medical, Shenzhen, China), together with circulating hormone concentrations and post-slaughter ovarian morphology. Transrectal ultrasonography was performed once daily on each of the 3 consecutive mornings preceding slaughter, immediately before blood collection at 08:00 h. The repeated examinations were used to support the consistency of ovarian status during the pre-slaughter observation period. Peripheral blood samples were collected immediately after each ultrasonographic examination at 08:00 h on the same 3 consecutive days. Serum hormone concentrations were used as supporting criteria for ovarian-state classification and were averaged across the three sampling days for subsequent statistical analysis. Based on ultrasonographic findings, post-slaughter ovarian morphology, endocrine profiles, and estrous observations, the animals were classified into five physiological or pathological groups: follicular phase, luteal phase, pregnancy, luteal cystic ovary, and inactive ovary. The follicular phase was defined by the consistent ultrasonographic identification of a dominant follicle and the absence of a functional corpus luteum during the 3-day observation period. After slaughter, the dominant follicle was supported by measuring its maximum diameter with a digital caliper, with follicles larger than 10 mm classified as dominant follicles. Relatively low serum P_4_ and high E_2_ concentrations were used as supporting endocrine criteria. The luteal phase was defined by the consistent presence of a functional corpus luteum on ultrasonographic examination, supported by post-slaughter ovarian morphology and the corresponding serum hormone profile. Pregnancy status was determined from the farm’s synchronized artificial insemination records and transrectal ultrasonographic examinations and was further supported after slaughter by the presence of a fetus in the uterus. The four pregnant cows were at approximately 3–4 months of gestation, corresponding to approximately 90–120 days after insemination, and were therefore classified as representing mid-gestation rather than early pregnancy [17,18]. Luteal cystic ovaries were defined by the consistent presence of a luteinized cystic structure with a maximum diameter greater than 25 mm during the three ultrasonographic examinations, together with disrupted estrous cyclicity. Inactive ovaries were diagnosed when no dominant follicle larger than 8 mm or functional corpus luteum was detected in any of the three consecutive ultrasonographic examinations, together with the absence of observable estrous behavior. Finally, 18 cows were included in the snRNA-seq analysis, comprising follicular-phase cows (*n* = 4), luteal-phase cows (*n* = 4), pregnant cows (*n* = 4), cows with luteal cystic ovaries (*n* = 3), and cows with inactive ovaries (*n* = 3). All animals were clinically healthy, maintained under comparable management conditions, and slaughtered as part of the farm’s routine production and culling procedures. No animal was slaughtered specifically for this study.

### 2.3. Sample Collection

Blood samples were collected from the coccygeal vein at 08:00 h on each of the 3 consecutive days before slaughter. Approximately 10 mL of blood was collected into BD Vacutainer plain serum tubes without anticoagulant (Becton, Dickinson and Company, Franklin Lakes, NJ, USA) and allowed to clot at room temperature for 30 min. The samples were subsequently centrifuged at 3000× *g* for 15 min at 4 °C. Serum was aliquoted and stored at −80 °C until hormone analysis. Before slaughter, the animals were rendered unconscious by electrical stunning in accordance with standard commercial procedures and animal-welfare regulations and were subsequently exsanguinated. No animals were slaughtered specifically for this study. Both ovaries were collected within 10 min after slaughter, rinsed with sterile saline, washed with ice-cold Dulbecco’s phosphate-buffered saline (DPBS; Biosharp, Beijing, China) to remove residual surface blood, and gently dried using sterile absorbent paper. The diameter of each follicle was measured at its maximum external diameter using a vernier caliper. Follicles with a diameter of less than 4 mm were classified as small follicles, whereas those with a diameter of 8 mm or greater were classified as large follicles. Two small follicles and, when present, two large follicles were sampled from each animal. The largest accessible follicle from each ovary was aspirated using a sterile syringe, and the collected follicular fluid was maintained on ice for subsequent ELISA analysis. Follicular fluid was not collected from inactive ovaries because large follicles were absent. Following follicular-fluid aspiration, the remaining follicular wall was retained for tissue sampling. Approximately 3-mm^3^ tissue fragments were dissected from multiple anatomical regions of each pair of ovaries, including the cortex and medulla and, when present, the corpus luteum, small follicles, and large follicles. Importantly, the sampled follicular tissues included the follicular wall and its GC layer, thereby allowing GC nuclei to be represented in the whole-ovary nuclear suspension. GCs were not separately isolated or enriched before nuclei isolation. Tissue fragments collected from the different anatomical regions of the same animal were pooled to generate a whole-ovary sample. All pooled tissues were immediately transferred into pre-cooled cryovials, snap-frozen in liquid nitrogen, and stored at −80 °C until nuclei isolation.

### 2.4. Hormone Determination

Concentrations of E_2_, FSH, luteinizing hormone (LH), and progesterone (P_4_) in both follicular fluid and serum were measured using bovine-specific ELISA kits (UpingBio, Hangzhou, China) according to the manufacturer’s instructions. The catalog number, assay method, analytical sensitivity, and intra- and inter-assay coefficients of variation for each kit are summarized in Supplementary Table S1. For each assay, 50 μL of the standard solution or sample was added to the designated wells. E_2_, P_4_, and FSH were quantified using competitive ELISA formats, whereas LH was measured using a sandwich ELISA. Plates were incubated at 37 °C for 1 h and subsequently washed five times. Horseradish-peroxidase-conjugated reagents and chromogenic substrates were then added, and the plates were incubated at 37 °C in the dark for 15 min. After addition of the stop solution, absorbance was measured at 450 nm using a microplate reader. Standard curves were fitted using a four-parameter logistic model, and hormone concentrations were calculated according to the corresponding standard curves. Samples with concentrations exceeding the upper limit of the detection range were diluted appropriately and reanalyzed, and the final concentrations were corrected using the corresponding dilution factors. Each biological sample was analyzed in technical triplicate, and the mean of the technical replicates was treated as one measurement for each animal. For serum samples, the hormone concentrations measured over the 3 consecutive sampling days were averaged to reduce the influence of day-to-day variation. However, this sampling scheme was not designed to characterize the pulsatile secretion patterns of FSH or LH.

### 2.5. Nuclei Isolation and Library Preparation

SnRNA-seq libraries were prepared using the DNBelab C Series Single-Nucleus Library Prep Kit (MGI, Shenzhen, China) according to the manufacturer’s instructions. Frozen pooled ovarian tissues from each animal were maintained on dry ice, mechanically minced, and homogenized on ice in Nuclei EZ Lysis Buffer (Sigma-Aldrich, St. Louis, MO, USA). During homogenization and lysis, nuclei were released from the different cellular components represented in the pooled ovarian tissues, including GC nuclei derived from the follicular wall. No GC-specific enrichment or sorting was performed before library construction. Therefore, GC nuclei were captured as part of the unsorted whole-ovary nuclear suspension together with nuclei from other ovarian cell populations. The homogenate was filtered through a 40-μm strainer, and nuclei were collected by centrifugation at 500× *g* for 5 min at 4 °C and resuspended in ice-cold DPBS. Nuclear concentration and integrity were evaluated using fluorescence microscopy, 0.4% Trypan Blue staining (Solarbio, Beijing, China), and Countess cell-counting chambers (Thermo Fisher Scientific, Waltham, MA, USA). Trypan Blue staining was used to facilitate visualization and counting of nuclei rather than to assess cell viability. Nuclear suspensions containing more than 80% morphologically intact nuclei, with minimal aggregation and debris, were retained for library construction. The nuclear concentration was adjusted to approximately 1000 nuclei/μL. Nuclei were encapsulated with barcoded gel beads using the DNBelab C4 microfluidic system to generate gel bead-in-emulsion droplets. Following emulsion disruption and bead recovery, barcoded cDNA was synthesized by reverse transcription, followed by second-strand synthesis and 14 cycles of cDNA amplification. Amplified cDNA fragments of 200–400 bp were purified and used for indexed library preparation with the DNBelab C4 Library Prep Module. The libraries were amplified using 10–12 PCR cycles, purified, and evaluated using the Qubit ssDNA Assay Kit (Thermo Fisher Scientific, Q10212). Qualified libraries were sequenced on the DNBSEQ-T7 platform in paired-end mode, with read lengths of 30 bp for read 1 and 100 bp for read 2.

### 2.6. snRNA-Seq Data Processing and Analysis

Raw snRNA-seq data were processed using dnbc4tools (v2.1.3). Briefly, raw FASTQ files were demultiplexed according to the DNBelab C-series library structure to extract cell barcodes and unique molecular identifiers (UMIs). High-quality reads were then aligned to the Bos taurus reference genome ARS-UCD1.2 using STAR (v2.7.2b) with default parameters [19]. After alignment, UMI collapsing was performed to remove PCR duplicates, and three primary output files—barcode, feature, and gene-cell count matrix were generated. All downstream analyses were conducted in R. snRNA-seq matrices from 18 cattle were integrated using Seurat (v5.3.0) [20]. Nuclei were retained based on the criteria nFeature_RNA > 750, nFeature_RNA < 6500, and percent.mt < 3, resulting in 158,819 nuclei. To further remove low-complexity, doublet, or apoptotic nuclei, doublet detection was performed using DoubletFinder (v2.0.3), yielding a final dataset of 154,054 high-quality nuclei [21]. After quality control, gene expression values were normalized using the “LogNormalize” method with a scaling factor of 10,000. Highly variable genes (*n* = 2000) were identified using the “vst” method, and dimensionality reduction was performed using PCA on the top 20 principal components. To account for potential batch effects and regional technical variation among ovarian samples, datasets were integrated using Seurat’s CCA-based integration framework followed by mutual nearest neighbor alignment, and batch effects were further mitigated using Harmony (v1.2.3) with default settings [22]. Finally, UMAP was computed using the top 20 principal components for visualization.

### 2.7. Cell Clustering and Cell Type Identification

The optimal clustering resolution was determined to be 0.7 using the clustree package (v0.5.1), after which the FindClusters function was applied to identify a total of 27 cell clusters [23]. Differentially expressed genes (DEGs) for each cluster were identified using the FindAllMarkers function with parameters min.pct = 0.25, logfc.threshold = 0.25, and adjusted *p*-value < 0.05. Cell clusters were subsequently annotated based on canonical marker genes reported in previous studies. Following whole-dataset clustering, GC nuclei were identified based on the coordinated expression of established GC markers. The identified GC nuclei were subsequently extracted from the whole-ovary dataset and subjected to GC-specific dimensionality reduction, reclustering, and subtype annotation.

### 2.8. High-Dimensional Weighted Gene Co-Expression Network Analysis

We performed high-dimensional weighted gene co-expression network analysis (hdWGCNA) using the hdWGCNA (v0.3.3) package to identify transcriptional modules within GCs across different physiological states [24]. To reduce noise and improve signal detection, metacells were first constructed by aggregating transcriptionally similar cells based on cell type identity. A signed co-expression network was then built, with the soft-thresholding power optimized to achieve approximate scale-free topology. Gene modules were identified via hierarchical clustering of the topological overlap matrix, and hub genes within each module were defined as those with high module membership. Module activity scores were calculated at single-cell resolution using UCell, enabling the assessment of module dynamics across cell populations.

### 2.9. Pseudotime Analysis

Pseudotime trajectory inference was performed using Monocle (v2.32.0) [25]. Seurat-processed nuclei were converted to a CellDataSet object, normalized, and filtered for highly variable genes (HVGs): genes with mean expression ≥ 0.1 and empirical dispersion ≥ 1× fitted dispersion were retained as HVGs and set as the ordering filter. Dimensionality reduction (DDRTree algorithm) and pseudotime ordering were implemented via orderCells. Genes with expression changes associated with pseudotime were identified using differentialGeneTest, with significance defined as *q*-value < 0.01. Branch-specific expression at branch point 1 was analyzed via Branch Expression Analysis Modeling, and genes with *q*-value < 1 × 10^−5^ were considered branch-associated. The top 100 such genes were visualized in a branched heatmap.

### 2.10. Cell-Cell Communication Analysis

Cell-cell communication was analyzed using CellChat (v1.6.1) [26]. A CellChat object was generated from the normalized expression matrix and cell type annotations. The CellChatDB.human database was used as the reference ligand-receptor database, and human ligand-receptor (L-R) pairs were mapped to bovine orthologs based on gene homology. Default preprocessing steps were applied to infer communication probabilities and signaling pathway activities. Interaction networks were visualized using CellChat’s built-in plotting functions and the patchwork (v1.3.0) package. Contribution and network centrality analyses were performed to identify key L-R pairs, major signaling senders and receivers, and dominant communication pathways.

### 2.11. Ligand-Target Regulatory Analysis

To identify potential ligands driving the phenotype of specific clusters, we employed the NicheNet (v2.2.1.1) package [27]. Ligands and receptors were filtered using the NicheNet ligand-receptor database (lr_network) and the ligand-target matrix. Differential expression in sender and receiver cells was computed with calculate_niche_de (type = “sender/receiver”), applying a minimum expression fraction of 10%, truncating infinite log_2_ fold changes, and retaining only bonafide = TRUE interactions. DE scores were processed with process_niche_de() and combined using combine_sender_receiver_de() with min_lfc as the ligand-receptor specificity metric. Downstream target genes in receiver cells were inferred using calculate_niche_de_targets() and process_receiver_target_de(), applying thresholds of LFC ≥ 0.5 or ≥ 1.0. The top 20 ligand-regulated targets were identified and presented per niche.

### 2.12. Functional Enrichment Analysis

To ensure species-specific functional interpretation, all Gene Ontology (GO) enrichment analyses were uniformly performed using the clusterProfiler package (v4.12.6) with the Bos taurus annotation database org.Bt.eg.db [28].

### 2.13. Single-Cell Flux Estimation Analysis

Metabolic fluxes at single-cell resolution were estimated using scFEA (v1.1.2) in Python [29]. The analysis was based on a curated bovine-specific metabolic network comprising 466 metabolic modules. Gene expression matrices from GCs were input to scFEA, which computes probabilistic flux distributions by integrating gene expression with the stoichiometric constraints of each module. Preprocessing included filtering lowly expressed genes and normalizing counts to account for cell-specific sequencing depth. The output provided predicted fluxes for each metabolic module, enabling downstream comparisons of metabolic activity across GC subtypes and physiological states.

### 2.14. Statistical Analysis

All statistical analyses were performed using R software (version 4.4.1). Data are presented as mean ± standard deviation (SD). Hormone concentrations and the E_2_/P_4_ ratio in both serum and follicular fluid were compared among different physiological states using one-way analysis of variance (ANOVA), followed by Tukey’s Honest Significant Difference (HSD) post hoc test for multiple pairwise comparisons. Normality of residuals and homogeneity of variances were assessed using the Shapiro-Wilk test and Levene’s test, respectively. Statistical significance was set at * *p* < 0.05, ** *p* < 0.01, and *** *p* < 0.001. Different superscript letters (a, b, c, d) within each row indicate significant differences between groups at *p* < 0.05.

## 3. Results

### 3.1. Comparative Analysis of Hormone Levels Across Physiological States

Serum hormone concentrations differed significantly among ovarian states (Table 1). E_2_ concentrations were highest during the follicular phase and lowest in cows with inactive ovaries, with pregnancy showing an intermediate level (*p* = 1.78 × 10^−8^). Significant overall differences were also observed for FSH and LH (*p* = 1.57 × 10^−3^ and *p* = 1.00 × 10^−3^, respectively), although several groups showed overlapping pairwise comparisons. Serum P_4_ differed markedly among groups (*p* = 2.80 × 10^−8^), reaching the highest concentration in cows with luteal cystic ovaries, followed by pregnant and luteal-phase cows, whereas follicular-phase and inactive cows showed relatively low concentrations.

Follicular-fluid hormone profiles also varied among ovarian states (Table 2). E_2_ concentrations were higher in follicular-phase and pregnant cows than in luteal-phase and luteal cystic cows (*p* = 9.95 × 10^−5^). FSH showed a modest overall difference (*p* = 0.019), with a higher concentration in the follicular phase than in the luteal cystic group, whereas LH did not differ significantly among groups (*p* = 0.182). Follicular-fluid P_4_ was markedly elevated in luteal cystic ovaries compared with the other states (*p* = 4.42 × 10^−8^). The E_2_-to-P_4_ ratio was highest in follicular-phase samples, lowest in luteal-phase and cystic samples, and intermediate during pregnancy (*p* = 0.020). Together, these results indicate a relatively estrogen-dominant profile during the follicular phase and pronounced progesterone predominance in luteal cystic ovaries.

### 3.2. Construction of a High-Resolution Single-Nucleus Atlas Across Five Ovarian States

**Figure 1:**
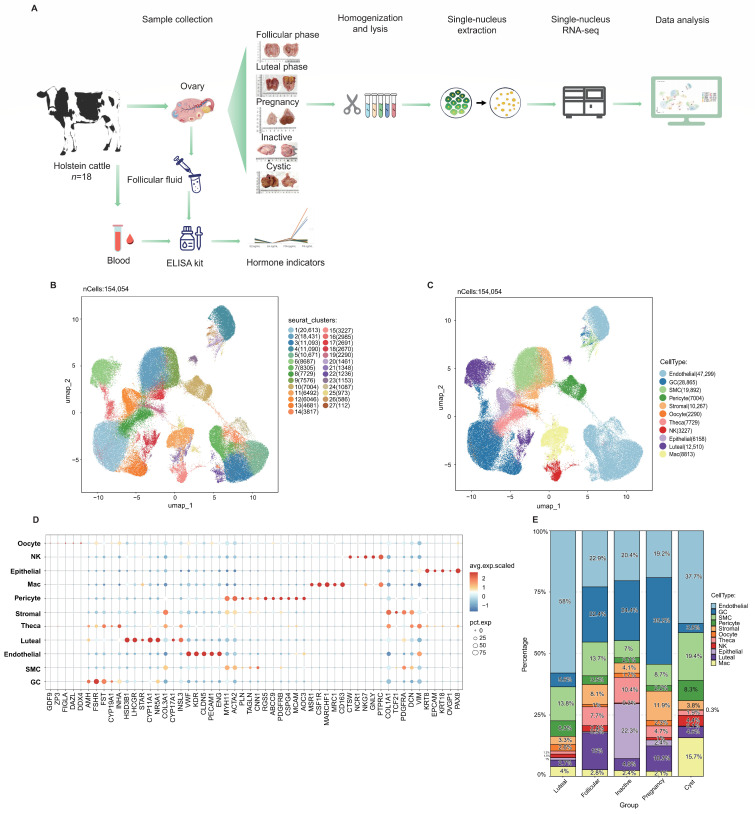
Single-nucleus transcriptome landscape of the Holstein cattle ovary. (**A**) Flowchart overview of Holstein cows. (**B**) UMAP projections identifying 27 specific clusters representing the ovary cell types. (**C**) UMAP projections showing eleven major ovarian cell types. (**D**) Marker gene expression levels and proportions across cell types. (**E**) Percentage of cell types in luteal phase, follicular phase, pregnancy, inactive and luteal cystic ovary.

SnRNA-seq was performed on 18 bovine ovarian samples representing five physiological states: follicular, luteal, pregnancy, cystic and inactive (Figure 1A). After applying strict quality filters and removing doublets, a total of 154,054 high-quality nuclei were retained (Supplementary Table S2). Each sample contributed approximately 5000–14,000 nuclei and expressed about 21,000–23,000 genes. Mean read depth ranged from 20,000 to 43,000 reads per nucleus. Sequencing quality was high, with mapping rates of 76–91% and Q30 values of 85–90%. After data integration, unsupervised clustering resolved 27 transcriptionally distinct clusters (Figure 1B). Nearly all clusters were detected across all physiological states, except cluster 27, which appeared only in luteal, inactive and pregnancy ovaries (Supplementary Figure S1A).

Based on previously published cell type-specific marker genes, we systematically annotated the major cell populations in ovarian tissues across five physiological states in Holstein cattle (Figure 1C). A total of 27 transcriptionally distinct clusters were identified and assigned to known ovarian cell types (Figure 1D, Supplementary Figure S1B). C1, C13, C16, and C26 were annotated as GCs, characterized by high expression of *AMH*, *FSHR*, *FST*, *CYP19A1*, and *INHA*. Smooth muscle cells (SMC) were identified in C2 and C20 based on the expression of canonical markers such as *MYH11*, *ACTA2*, *PLN*, *TAGLN*, and *CNN1*. Endothelial cells, defined by robust expression of *VWF*, *KDR*, *CLDN5*, *PECAM1*, and *ENG*, were distributed across C3, C4, C5, C7, C14, C22, and C24. C6, C18, and C23 were annotated as luteal cells, exhibiting enriched expression of key steroidogenic genes, including *HSD3B1*, *LHCGR*, *STAR*, *CYP11A1*, and *NR5A1*. The theca cell population was represented by C8 and was identified by the expression of *COL3A1*, *CYP17A1*, and *INSL3*. Stromal cells (C9 and C17) were characterized by extracellular matrix and mesenchymal-associated markers such as *COL1A1*, *TCF21*, *PDGFRA*, *DCN*, and *VIM*. C10 was annotated as pericytes based on the expression of *RGS5*, *ABCC9*, *PDGFRB*, *CSPG4*, *MCAM*, and *AOC3*. Immune cell populations were also clearly delineated. C11, C21, and C25 were identified as macrophages (Mac), marked by the expression of *MSR1*, *CSF1R*, *MARCHF1*, *MRC1*, and *CD163*. Natural killer (NK) cells were confined to C15 and expressed characteristic cytotoxic markers, including *CTSW*, *NCR1*, *NKG7*, *GNLY*, and *PTPRC*. Epithelial cells were annotated in C12 and C27 based on high expression of *KRT8*, *EPCAM*, *KRT18*, *OVGP1*, and *PAX8*. In addition, C19 exhibited a distinct transcriptional signature marked by *GDF9*, *ZP3*, *FIGLA*, *DAZL*, and *DDX4*, and was therefore annotated as oocytes. All cell types exist in various states. Collectively, these results define the cellular composition of the bovine ovary across multiple physiological states and provide a robust foundation for subsequent cell type-specific and state-dependent analyses.

Stacked proportion analysis showed marked state-dependent remodeling of the ovarian microenvironment (Figure 1E). Endothelial cells were enriched in luteal ovaries, whereas GCs expanded in follicular, inactive, and especially pregnancy ovaries, but declined sharply in cystic ovaries. Luteal cells were relatively abundant in follicular and pregnancy groups, stromal and SMC populations increased in pregnancy and cystic states, and Mac were most prominent in cystic ovaries. Oocytes remained rare across all groups. Together, these data indicate that ovarian state is accompanied by substantial reorganization of the cellular landscape, with the most conspicuous changes occurring in the GC and immune compartments.

### 3.3. Granulosa Cell Heterogeneity Across Ovarian States

**Figure 2:**
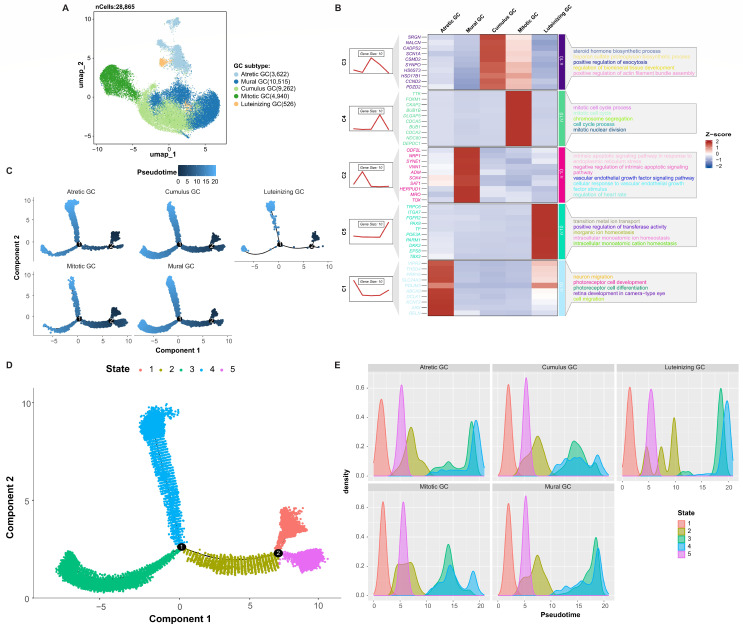
Single-nucleus transcriptome analysis of ovarian GCs. (**A**) UMAP projections of 28,865 GCs revealing five GC subtypes, including Mitotic GC, Cumulus GC, Luteinizing GC, Mural GC, and Atretic GC. (**B**) Functional enrichment based on the top 10 signature genes of each GC subtype, showing representative biological processes. (**C**) Pseudotime trajectory showing inferred transcriptional trajectory of five GC subtypes. (**D**) DDRTree trajectory arranging GCs into five pseudotime states with two inferred branching nodes. (**E**) Density distributions of the five GC subtypes along the inferred pseudotime trajectory.

Building upon the initial clustering of GCs, we performed further dimensionality reduction and re-clustering at a resolution of 0.9, yielding 16 distinct cell clusters. These clusters were systematically annotated into known GC subtypes (Figure 2A), based on the expression of established marker genes and Spearman coefficient (Supplementary Figure S1C,D). C0, C5, and C9 were collectively annotated as Cumulus GCs, based on their high expression of *CCND2*, *FST*, and *SRGN*. C4, C11, and C12 were identified as Atretic GCs due to their elevated expression of *KCNT2*, *SLC24A3*, and *VIPR2*. The mural GC identity was assigned to C1, C2, C3, C8 and C15, which exhibited strong expression of mural GC markers *MEI4*, *ADM*, and *NRP1*. C6, C7, and C10 displayed high expression of proliferation-associated genes *TTK*, *CENPE*, and *CKAP2*, and were accordingly annotated as Mitotic GCs. Finally, C13 and C14 were classified as Luteinizing GCs based on their specific expression of markers *PAX8*, *PARM1*, and *ITGA7*. Functional annotation of subtype-specific DEGs suggested clear specialization: mitotic GCs were associated with cell-cycle programs, atretic GCs with apoptosis and stress responses, mural GCs with steroidogenesis and extracellular matrix (ECM) remodeling, and other clusters with ion transport, structural organization, and differentiation-related processes (Figure 2B). The relative abundance of GC subtypes changed across physiological states (Supplementary Figure S1E). Mural GCs were the dominant subtype in all groups and were highest in pregnancy and cystic ovaries. Cumulus GCs were enriched in the follicular phase and inactive ovaries, while mitotic GCs were relatively more common in follicular and inactive samples. Atretic GCs were increased in luteal and cystic ovaries, whereas luteinizing GCs remained a minor population with slightly higher proportions in luteal and cystic states. These findings suggest that state-associated differences in the relative representation of GC subtypes.

To examine the transcriptional relationships among GC subtypes across physiological and pathological ovarian states, we performed Monocle2-based pseudotime analysis on 28,865 GCs (Figure 2A). The analysis arranged the cells along a branched inferred transcriptional trajectory (Figure 2C,D). Because the cells were collected cross-sectionally from different animals and ovarian states, this trajectory represents a relative ordering of transcriptional states rather than actual developmental time. The five GC subtypes occupied different regions of the inferred trajectory. Atretic GCs were predominantly located at lower pseudotime values, whereas mural and luteinizing GCs were more frequently represented at higher pseudotime values. Cumulus and mitotic GCs were mainly distributed at intermediate pseudotime positions (Figure 2E). These distributions suggest transcriptional relationships among GC states but do not suggest direct developmental transitions between the identified subtypes. When the inferred trajectory was stratified by ovarian state, its overall topology was broadly similar, although the distributions of cells along pseudotime differed among groups (Supplementary Figure S1F). Pregnancy and luteal cystic ovaries showed a higher representation of cells at higher pseudotime values, whereas follicular and inactive ovaries contained more cells at intermediate pseudotime positions. These patterns represent state-associated differences in transcriptional distribution along the inferred trajectory.

**Figure 3:**
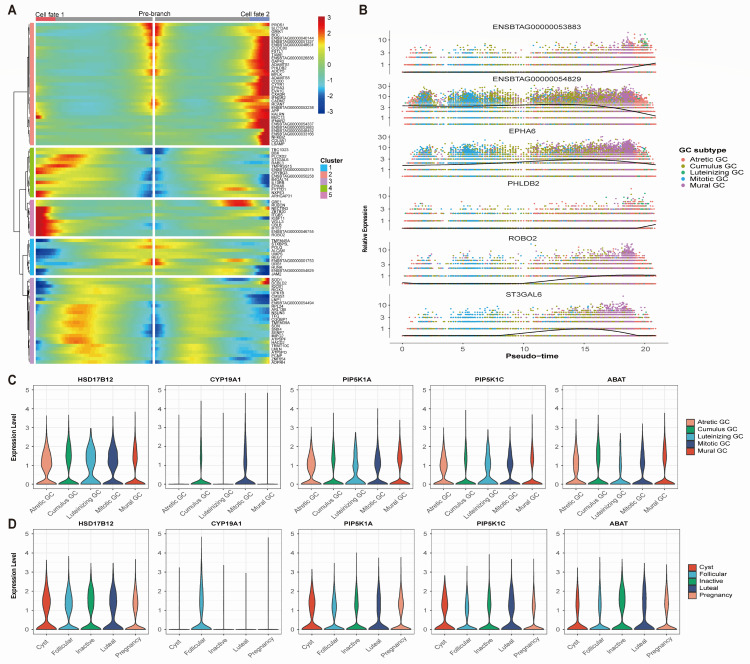
Pseudotime-associated transcriptional patterns and key gene expression in GCs. (**A**) Pseudotime-associated gene-expression patterns across the pre-branch and two inferred branches, showing representative branch-associated genes. (**B**) Expression heatmap of the most significantly dynamic genes along the inferred pseudotime trajectory across five GC subtypes. (**C**) Expression levels of selected functional genes across five GC subtypes. (**D**) Expression levels of selected genes across ovarian states.

To identify genes associated with the inferred branch structure, we performed branched expression analysis modeling (BEAM) at branch point 1 of the pseudotime trajectory. This analysis identified 6018 branch-dependent genes, which were grouped into five expression modules according to their pseudotime-associated expression patterns (Figure 3A). GO enrichment analysis revealed that each module was associated with specific biological processes, reflecting transcriptional programs associated with the inferred GC states and branches (Supplementary Table S3). Cluster 1 was enriched in metabolic and biosynthetic processes, with key genes including ribosomal proteins (*RPL10*, *RPS26*) and mitochondrial components (*COX5B*, *UQCRQ*), indicating active protein synthesis and energy production. Cluster 2 was associated with cell adhesion and structural remodeling, marked by *ITGA9*, *THBS1*, and *NCAM1*, suggesting dynamic extracellular matrix interactions. Cluster 3 showed enrichment in autophagy and stress-related pathways, with representative genes such as *ULK1* and *ATG13*, reflecting catabolic and adaptive responses. Cluster 4 was linked to morphogenesis and vascular signaling, highlighted by *VEGFA* and *PLXNA2*, implying roles in tissue remodeling and angiogenesis. Cluster 5 represented a proliferative population, characterized by cell cycle regulators including *MKI67*, *TOP2A*, and *CCND2*.

Mural GC markers, such as *MEI4*, showed a rapid increase in early pseudotime and maintained relatively stable expression at intermediate and higher pseudotime values. In contrast, genes involved in cell adhesion and structural remodeling, including *LAMA1*, *FAT3*, and *TENM2*, displayed a consistent upward trend, reaching their highest expression near the high-pseudotime end of the inferred trajectory. *NR4A2* and *ALDH1A2* also showed higher expression at higher pseudotime values, suggesting an association with distinct GC transcriptional states. Branch-specific analysis further highlighted *EPHA6*, *ROBO2*, *PHLDB2*, and *ST3GAL6*, together with two unannotated bovine genes, as highly dynamic candidate genes associated with the inferred branch structure (Figure 3B). In parallel, genes linked to steroidogenesis, phosphoinositide signaling, and GABA metabolism, including *HSD17B12*, *CYP191A1*, *PIP5K1A*, *PIP5K1C*, and *ABAT*, showed state-specific expression patterns, supporting a close connection between GC differentiation and metabolic regulation (Figure 3C,D).

### 3.4. State-Specific Granulosa Cell Co-Expression Networks Across Ovarian States

**Figure 4:**
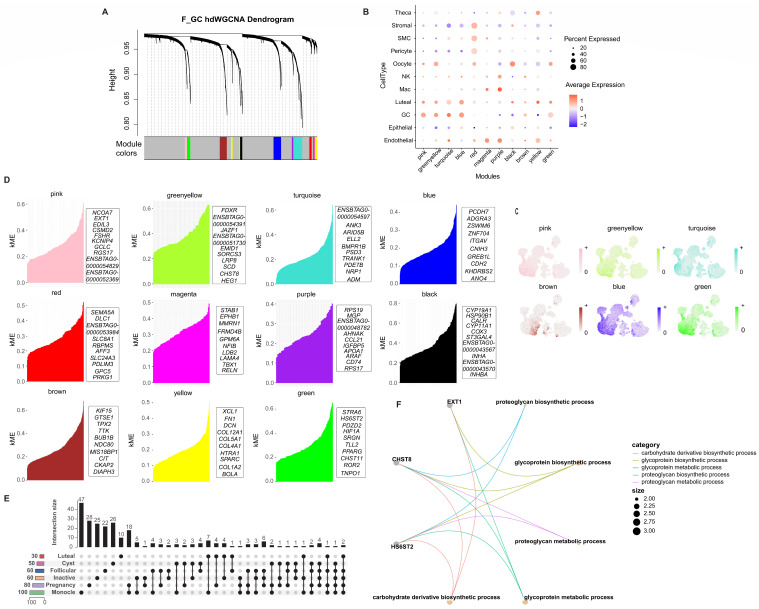
HdWGCNA reveals follicular phase GC-specific co-expression modules and functional enrichment. (**A**) Hierarchical clustering dendrogram of GC genes constructed using hdWGCNA with distinct gene modules indicated by different colors. (**B**) Dot plot showing cell-type specificity of major GC-associated modules in ovarian cell populations. (**C**) UMAP feature plots showing the expression distribution of the six GC-most-relevant modules. (**D**) Top 10 hub genes of representative GC-related modules ranked by kME value. (**E**) Intersection analysis between hdWGCNA module genes and Monocle-derived HVGs across follicular and other ovarian states. (**F**) GO enrichment analysis of hdWGCNA module genes.

HdWGCNA was applied to GC populations from follicular, luteal, pregnancy, inactive, and cystic ovaries to identify state-specific co-expression networks (Figure 4A). In follicular ovaries, 11 co-expression modules were identified, among which the pink, greenyellow, turquoise, brown, and green modules showed the strongest correlations with GCs (Figure 4B,C). Notably, the pink module contained the canonical GC marker *FSHR*, supporting the biological relevance of the network structure (Figure 4D). Intersection of the top 10 hub genes by kME from these GC-associated modules with the top 100 HVGs identified by Monocle pseudotime analysis yielded 4 shared genes, together with 22 hdWGCNA-specific genes (Figure 4E). These genes were significantly enriched in proteoglycan and glycoprotein biosynthetic and metabolic processes, as well as carbohydrate derivative biosynthesis. These findings suggest that the follicular phase is characterized by an intensified synthesis of ECM components, which likely facilitates follicular structural expansion (Figure 4F). In luteal ovaries, 9 modules were identified. (Supplementary Figure S2A). There were 7 genes that overlapped between hdWGCNA hub genes and Monocle HVGs, whereas 10 genes were uniquely identified by hdWGCNA (Figure 4E). These luteal-specific genes were *PRKAR2B*, *TCF4*, and *CAPRIN2*, suggesting a relatively stable endocrine-active state. Notably, *ARID5B* was shared between follicular and luteal GCs, suggesting a potential role in maintaining normal ovarian cyclicity (Figure 4E). During pregnancy, hdWGCNA identified multiple GC-associated modules (Supplementary Figure S2B), whose hub genes partially overlapped with Monocle-derived HVGs, yielding 18 shared genes, while 28 genes were uniquely captured by hdWGCNA (Figure 4E). Hub genes such as *LHCGR*, *KIF11*, and *CENPF* suggest active hormonal signaling and structural remodeling, supporting sustained luteal function and endocrine activity.

In contrast, inactive and cystic ovaries exhibited distinct but related transcriptional features indicative of dysregulated follicular development. In inactive ovaries, seven modules were observed (Supplementary Figure S2C). Five genes overlapped with Monocle HVGs, whereas 25 genes were uniquely identified by hdWGCNA (Figure 4E). These genes were enriched in chromosome condensation, sister chromatid segregation, nuclear chromosome segregation, chromosome segregation, nuclear division, organelle fission, and chromosome organization (Supplementary Figure S2D). Representative genes such as *TOP2A*, *NCAPG*, and *SMC4* suggested aberrant proliferative activity without normal differentiation. Similarly, cystic ovaries exhibited enrichment of genes involved in metabolic regulation and steroidogenesis, including *CPT1B*, *HSD3B1*, and *RORA*, pointing to altered metabolic and endocrine states. Importantly, shared genes between inactive and cystic ovaries, including *SAMD12*, *FGF12*, and *SYNPO2*, suggest common mechanisms underlying impaired follicular maturation and defective ovulation (Figure 4E). In addition, the overlap between follicular and cystic ovaries included key regulatory genes such as *FSHR* and *HIF1A*, indicating that cystic conditions may partially retain follicular-like regulatory features while deviating toward pathological states (Supplementary Table S4). In the cystic ovary, GC-associated modules were identified, among which the black, blue, brown, turquoise and pink modules showed strong correlations with GC (Supplementary Figure S2E). In contrast, 26 genes were uniquely identified by hdWGCNA, reflecting cyst-specific transcriptional coordination not captured by pseudotime analysis alone (Supplementary Table S4). These genes were significantly enriched in pathways associated with fatty acid metabolism, inflammatory response, retinoic acid receptor signaling, and peroxisome-related transport and protein localization, highlighting metabolic dysregulation and inflammatory features characteristic of cystic pathology (Supplementary Figure S2F).

### 3.5. Predicted Cell-Cell Communication During Ovarian State Transitions

**Figure 5:**
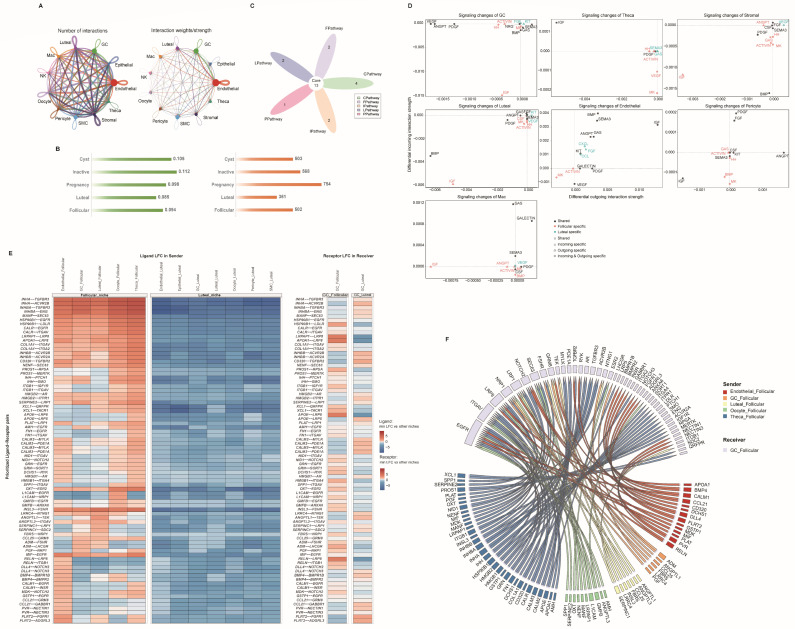
Global and state-specific remodeling of ovarian cell-cell communication networks. (**A**) Overview of cell-cell communication networks among all major ovarian cell types across the five physiological states. Edges represent predicted ligand-receptor interactions, and edge thickness indicates interaction strength. (**B**) Venn diagram summarizing signaling pathways commonly shared across the five ovarian states, highlighting conserved intercellular communication programs. (**C**) Quantification of total interaction strength and number of inferred interactions in each ovarian state. (**D**) Comparison of incoming and outgoing signaling pathways between follicular and luteal ovaries. (**E**) NicheNet analysis of the follicular ovary, displaying differentially expressed ligands in sender cell types and corresponding target gene expression changes in GCs. (**F**) Circos plot of prioritized ligand-receptor pairs in the follicular ovary.

Given the pronounced state-specific GC transcriptional networks identified by hdWGCNA, we asked whether these intrinsic programs are accompanied by coordinated changes in intercellular communication across ovarian states (Figure S3A). We next performed CellChat analysis to systematically characterize cell-cell signaling networks across ovarian physiological states. We first assessed global intercellular communication landscapes across ovarian states using CellChat (Figure 5A). Pregnancy ovaries showed the most complex interaction landscape, whereas luteal ovaries had the fewest inferred interactions. Inactive ovaries showed globally reduced outgoing and incoming signaling, while cystic ovaries retained fewer but stronger communication axes, together with a shift toward inflammatory and stress-associated pathways (Figure 5B). We next classified signaling pathways into conserved and state-specific categories. Thirteen signaling pathways were shared across all ovarian states, including IGF, BMP, PDGF, VEGF, FGF, ANGPT, and related growth factor networks (Figure 5C). These patterns indicate progressive rewiring of the ovarian communication network across physiological and pathological states (Supplementary Table S5).

To further dissect how intercellular signaling is remodeled during ovarian state transitions, we compared differential outgoing and incoming interaction strengths between follicular ovaries and other physiological or pathological states. Across all comparisons, growth factor-associated pathways, including IGF, PDGF, VEGF, FGF, and BMP signaling, constituted the major contributors to differential communication, highlighting their central role in follicular-stage ovarian signaling networks (Figure 5D). During physiological transitions from the follicular phase to luteal or pregnancy states, GCs and theca cells exhibited a coordinated reduction in outgoing signaling strength, particularly in growth and proliferation-related pathways (Supplementary Figure S3B,C). These changes were accompanied by relatively modest alterations in incoming signaling, suggesting an orderly reprogramming of communication networks as follicles exit the growth phase. In contrast, inactive ovaries displayed a global attenuation of both outgoing and incoming signaling across multiple cell types, indicative of a collapsed and poorly coordinated communication landscape (Supplementary Figure S3D). Although a limited number of pathways, such as SPP1 and NRG-associated signaling, remained detectable, these signals appeared insufficient to sustain normal follicular activity. Notably, cystic ovaries exhibited a distinct pattern characterized by pronounced suppression of GC-derived outgoing signaling coupled with selective enhancement of inflammatory and metabolic stress-associated pathways, including TNF, VISFATIN, RESISTIN, and PARs signaling (Supplementary Figure S3E). These pathways primarily originated from immune and stromal compartments, indicating a shift away from GC-centered communication toward a pathological signaling milieu.

L-R analysis further showed that the dominant signaling axes were remodeled in a state-specific manner. Follicular-to-pregnancy transitions were characterized by *ANGPT*-*TEK*, *VEGF*-*VEGFR*, and *PTN*/*MDK* related interactions, consistent with vascular and structural remodeling (Supplementary Figure S4A,B). In inactive and cystic ovaries, immune-related pairs such as *GAS6*-*TAM*, *LGALS9*-*CD44*/*CD45*, and *CX3CL1*-*CX3CR1* became more prominent (Supplementary Figure S4C). Cystic ovaries additionally showed activation of *TNF*-*TNFR*, *NAMPT*-*INSR*, and *RETN*-*CAP1*, highlighting that inflammatory and metabolic cues may help drive the pathological niche (Supplementary Figure S4D).

### 3.6. Predicted Microenvironment-Driven Rewiring of Granulosa Cell Regulatory Programs

To further dissect how intercellular signaling is remodeled during ovarian state transitions, we performed NicheNet analysis by defining state-specific ovarian niches as signal senders and corresponding GC populations as receivers. Across all comparisons, revealed pronounced differences in ligand composition, regulatory potential, and downstream transcriptional programs, reflecting distinct GC regulatory strategies aligned with physiological demand and pathological disruption. In the follicular stage, GC regulatory programs were characterized by a relatively focused set of high-priority ligands with strong regulatory potential and positive activity, including *INHBA*, *INHA*, *AMH*, *SPP1*, *BMP4* and *OXT* (Figure 5E). These ligands exhibited high scaled expression and regulatory potential, indicating their dominant roles in shaping GC transcriptional states during follicular growth. Predicted target genes downstream of follicular stage ligands were enriched for estrogen biosynthesis, cell cycle progression, and ECM remodeling, including *CYP19A1*, *HSD17B1*, *CDK1*, *CCNB2*, *FOXM1* and *BIRC5*, supporting active GC proliferation and estrogen production (Figure 5F). Additional targets involved in lipid metabolism and structural expansion, such as *APOA1*, *APOE*, *COL1A1*, and *COL1A2*, were consistent with the metabolic and architectural demands of growing follicles.

In contrast, GC_Luteal cells exhibited a broader and more complex ligand landscape. High-priority ligands included *PDGFD*, *PDGFA*, *IGF1*, *ANGPT1*, *HGF*, *SEMA5A*, *DKK2*, and *WNT3A*, many of which displayed elevated regulatory potential and ligand activity (Supplementary Figure S5A). Correspondingly, predicted target genes were enriched for angiogenesis, anti-apoptotic signaling, and progesterone synthesis, including *PDGFB* and *TIE1* (vascular stabilization), *BCL2* and *VAV2* (cell survival via PI3K-AKT signaling), and *WT1* and *TGFBR2*, which are implicated in steroidogenic regulation (Supplementary Figure S5B). Genes involved in calcium signaling and ion transport, such as *CACNB2* and *TRPC4*, further supported calcium-dependent progesterone secretion. Pregnancy-associated GCs displayed a distinct set of high-priority ligands related to steroidogenic support and metabolic adaptation, including *IGF1*, *BMP4*, *BMP7*, and *FGF9*, all of which showed positive ligand activity and regulatory potential (Supplementary Figure S5C). These ligands are known activators of PI3K-AKT and SMAD pathways, promoting progesterone biosynthesis and metabolic adaptation. Consistently, predicted downstream target genes included regulators of luteal function and hormone production, such as *KLF9*, *DEPP1*, and *PTGES2*, indicating reinforcement of progesterone synthesis (Supplementary Figure S5D). In addition, pregnancy-specific ligand inputs prominently included immune-modulatory factors such as *SERPING1*, *C3*, *A2M*, and *LGALS3*, whose predicted targets were enriched for immune tolerance and anti-inflammatory functions. This pattern suggests active protection of the pregnancy-maintaining corpus luteum from immune-mediated regression.

GC_Inactive were characterized by a markedly expanded set of prioritized ligands, including *HPSE*, *MYRIP*, *WT1*, *GSTA5*, *CACNB2*, *SNAP91*, *ENPP1*, *FGFR1*, and *EPHA7* (Supplementary Figure S5E). Predicted target genes were enriched for cell survival, stress resistance, metabolic homeostasis, and suppression of aberrant activation, rather than proliferation or differentiation. Key regulatory nodes included *WT1*, as well as *FGFR1* and *NTRK2*, which are associated with pro-survival and repair-related signaling (Supplementary Figure S5F). Despite pronounced state-specific differences, subset of ligands—such as *MMP9*, *SPP1*, *TTR*, *WNT10A*, *OCLN*, and *VEGFB*—was conserved between follicular and inactive niches, suggesting a basal signaling framework required for GC maintenance regardless of functional state. In contrast to physiological ovarian states, cystic GCs exhibited profoundly altered ligand-target landscape dominated by excessive inflammatory signaling. Cystic niches were enriched for pro-inflammatory ligands, including *IL1B*, *TNF*, *CCL2*, *CCL4*, *CCL8*, *CXCL2*, *CXCL10*, and *CXCL11* (Supplementary Figure S5G). These inflammatory cues were accompanied by increased expression of immune adhesion and interaction ligands, indicating enhanced immune cell recruitment and retention (Supplementary Figure S5H). Ligands involved in dysregulated proliferation and survival, including *TGFB1*, *ADAM17*, *FGF7*, *NAMPT*, and *TGFA*, further suggested breakdown of normal GC proliferative control. High-priority regulatory hubs, such as *PTPRT*, *SEMA4D*, *LGALS3*, and *GRN*, exhibited the strongest regulatory potential in cystic GCs. Predicted target genes were related to NF-κB-mediated inflammatory signaling, immune regulation, and structural disruption, reflecting persistent inflammation, impaired extracellular matrix integrity, and loss of follicular homeostasis.

### 3.7. Predicted Metabolic Reprogramming of Granulosa Cell Subtypes

**Figure 6:**
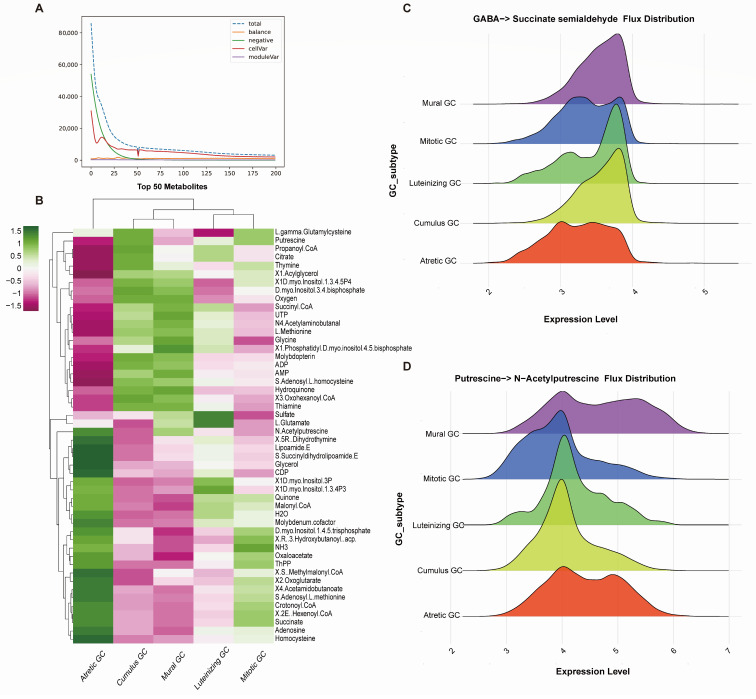
Metabolic reprogramming of GC subtypes inferred by scFEA. (**A**) Convergence of the scFEA model during iterative optimization. (**B**) Heatmap of the top 50 differentially abundant metabolites in each GC subtype. (**C**) Flux distribution of GABA to succinate semialdehyde across five GC subtypes. (**D**) Flux distribution of putrescine to N-acetylputrescine across five GC subtypes.

To further elucidate the functional consequences of microenvironment-driven regulatory programs, we applied scFEA to infer metabolic flux alterations across five GC subtypes. We first examined the variance decomposition of inferred metabolic activities (Figure 6A). The total variance was primarily attributable to cellVar, with a substantial but smaller contribution from metabolic moduleVar. This pattern indicates that metabolic heterogeneity among GCs is driven by both intrinsic cell-to-cell differences and coordinated regulation of metabolic modules, rather than by stochastic noise.

Analysis of the top 50 differential metabolites revealed clear subtype specific metabolic clustering (Figure 6B). Intermediates of the TCA cycle, including citrate, succinate, oxaloacetate, and succinyl-CoA, showed differential distribution across GC subtypes. Cumulus GCs were enriched in citrate, UTP, putrescine, oxygen, and molybdopterin, whereas mural and luteinizing GCs showed higher levels of succinyl-CoA, propanoyl-CoA, acylglycerol, and related lipid derivatives. Atretic GCs were characterized by accumulation of oxaloacetate, malonyl-CoA, crotonoyl-CoA, hexenoyl-CoA, adenosine, N-acetylputrescine, homocysteine, SAM, quinone, and IP3, together with reduced PIP2, suggesting impaired energy coupling, lipid turnover, and redox balance.

At the physiological state level, follicular and luteal phases were enriched for ovulation-related metabolites, including TCA intermediates and lipid-associated compounds, whereas pregnancy showed increased polyamine-related metabolites and inactive/cystic states displayed disrupted nucleotide and redox profiles (Supplementary Figure S6A). The GABA-to-succinate semialdehyde pathway was reduced activity in atretic GC despite broad cellular representation, suggesting impaired energy coupling (Figure 6C). In contrast, the conversion of putrescine to N-acetylputrescine was broadly active but reached the highest flux in mural GC, highlighting active polyamine metabolism associated with ovulation-related processes (Figure 6D). Consistent with these findings, pathway-level analysis revealed that several ovulation-related metabolic reactions were selectively activated in specific GC subtypes (Figure S6B). Phosphoinositide metabolism was enriched in atretic GC, whereas ascorbate biosynthesis was restricted to mural GC. Fatty acid-related pathways were preferentially active in atretic, mitotic, and luteinizing GC, but were less active in mural and cumulus GC. In contrast, pathways such as GM3 to GD3 conversion and ubiquinone biosynthesis showed uniformly low activity across all subtypes. Collectively, these results may suggested that ovulation-related metabolic reprogramming spans TCA cycle activity, lipid metabolism, nucleotide turnover, polyamine metabolism, and phosphoinositide signaling, while metabolic disruption is associated with follicular atresia and ovarian dysfunction.

To further link metabolic reprogramming with transcriptional regulation, we examined key genes corresponding to ovulation-associated metabolic pathways (Supplementary Table S6). Notably, several genes were commonly enriched in both mural and luteinizing GC, including lipid metabolism-related genes, steroidogenic enzymes, and phosphoinositide signaling regulators. In addition, *ABAT*, involved in GABA metabolism, was also detected, consistent with the observed metabolic flux changes. Importantly, these genes were previously identified as dynamically regulated along the GC differentiation trajectory, suggesting coordinated regulation of metabolism and cell fate during ovulation.

## 4. Discussion

This study provides a single-nucleus transcriptomic atlas of the bovine ovary across follicular, luteal, pregnancy, inactive, and luteal cystic states, characterizing state-associated differences in GC heterogeneity, ovarian microenvironmental signaling, and predicted metabolic programs. The hormone profiles provided complementary endocrine information for classifying the ovarian states. The low serum E_2_ and P_4_ concentrations in cows with inactive ovaries were consistent with the absence of an active dominant follicle and functional luteal tissue and may be associated with insufficient gonadotropin support under altered nutritional or metabolic conditions [30,31]. In contrast, the elevated serum and follicular-fluid E_2_ concentrations during the follicular phase were consistent with the steroidogenic activity of a dominant follicle [32]. The markedly elevated serum and follicular-fluid P_4_ concentrations in the luteal cystic group supported the presence of persistent luteinized tissue with sustained progesterone secretion [33].

The follicular-fluid E_2_/P_4_ ratio provided additional information on the relative estrogenic and progestogenic characteristics of the follicular environment. Previous bovine studies have commonly used an E_2_/P_4_ ratio greater than 1 to define estrogen-active follicles, whereas lower values are associated with reduced estrogenic activity, luteinization, or follicular atresia [34]. In the present study, the follicular group had the highest E_2_/P_4_ ratio, consistent with an estrogen-dominant follicular environment. In contrast, the very low ratio in luteal cystic ovaries reflected pronounced progesterone predominance, while the pregnancy group showed an intermediate profile. The FSH and LH measurements require more cautious interpretation. Both gonadotropins are primarily secreted by the anterior pituitary, and their circulating concentrations vary over time. In cattle, LH and FSH secretion includes pulsatile components, and studies designed to characterize these patterns have used blood-sampling intervals of approximately 5–20 min rather than isolated daily samples [35]. In the present study, blood was collected at the same time on three consecutive days and the measurements were averaged, which reduced the influence of variation associated with a single sampling day but did not resolve pulse frequency, pulse amplitude, or complete systemic gonadotropin dynamics. Accordingly, the observed differences in serum FSH and LH should be interpreted as average concentrations at the sampled time points rather than as comprehensive measures of pituitary secretion. This limitation may partly explain the overlapping pairwise comparisons among groups and the relatively high serum LH concentration observed in the inactive group. Classic cattle studies suggest that both LH and FSH show pulsatile secretion and that high-frequency sampling is required to resolve these patterns [35].

Detection of FSH and LH in follicular fluid does not by itself suggest their local ovarian synthesis. Follicular fluid is partly formed by movement of fluid from the thecal vasculature, and even relatively large serum-derived proteins may enter the follicular compartment [36,37]. Therefore, follicular-fluid FSH and LH may largely reflect transfer from the systemic circulation, followed by local retention, receptor binding, metabolism, or degradation within the follicle. Previous work has directly detected FSH and LH in bovine follicular fluid and reported altered follicular-fluid gonadotropin concentrations in cows with chronic cystic ovarian disease [38].

The cellular atlas expands previous ovarian single-cell studies by comparing several physiological and pathological states within an analytical framework. The identification of GCs, theca cells, luteal cells, stromal cells, endothelial cells, and immune populations was consistent with previous ovarian transcriptomic atlases across species [10,39]. GC abundance patterns across states—prominent in pregnancy and follicular phases but reduced in cystic ovaries—are consistent with known roles of follicular recruitment and the degenerative changes accompanying cystic ovarian disease [4,40]. Immune cell expansion, particularly macrophages, in cystic ovaries supports the view that chronic intra-ovarian inflammation contributes to cyst persistence [41]. Endothelial cell enrichment in luteal ovaries further aligns with the angiogenic demands of luteal tissue maintenance [42].

GC subclustering identified mitotic, cumulus, mural, atretic, and luteinizing transcriptional states, extending the conventional mural-cumulus classification. Monocle analysis arranged these nuclei along an inferred branched transcriptional trajectory. Because the samples were collected cross-sectionally from different animals and ovarian states, pseudotime does not represent chronological biological time, direct lineage progression, or experimentally observed transitions between GC subtypes. Atretic GCs were preferentially located at lower pseudotime positions, whereas mural and luteinizing GCs were more frequently represented at higher pseudotime positions, with cumulus and mitotic GCs distributed mainly at intermediate positions. These patterns indicate transcriptional relationships among GC states but do not suggest that one identified subtype develops directly into another. Similar GC heterogeneity and disease-associated changes in ovarian cell-state distributions have been reported in murine, primate, and pathological ovarian single-cell studies [43,44,45]. The association of cystic-ovary GCs with higher pseudotime positions may therefore reflect a distinct transcriptional state related to luteinization or persistence rather than actual late developmental time [46].

Co-expression network analysis revealed distinct regulatory programs across states. The ECM-centered gene program in follicular ovaries, with hub genes including *FSHR*, is consistent with proteoglycan synthesis requirements during antrum expansion [12,47]. Inactive ovaries were characterized by enrichment of cell cycle-related genes such as *TOP2A* and *NCAPG*, suggesting aberrant proliferative activity that fails to progress toward differentiation, a pattern consistent with continued follicle recruitment but insufficient LH signaling [48]. Cystic ovaries displayed features of metabolic reprogramming and inflammatory signaling. The shared presence of *HIF1A* in both follicular and cystic modules suggests that cysts may retain certain follicular features while existing in a hypoxic or pseudo-hypoxic state [49]. The overlap of *SAMD12*, *FGF12*, and *SYNPO2* between inactive and cystic states may point to shared cytoskeletal or organizational defects [50].

Intercellular communication analysis identified several signaling pathways—including IGF, BMP, and VEGF—that were shared across all states examined, suggesting roles in basal ovarian homeostasis [51,52]. The global attenuation of signaling in inactive ovaries suggests that impaired follicular maturation involves not only intrinsic GC deficits but also reduced niche-derived support, likely reflecting diminished hypothalamic-pituitary input [53]. Cystic ovaries showed a distinct pattern: GC-derived signals were reduced, while inflammatory and metabolic stress pathways, including TNF, VISFATIN, and RESISTIN, were enhanced [50,54]. Adipokine signaling via *NAMPT*-*INSR* and *RETN*-*CAP1* suggests that local metabolic signals may further disrupt follicular function [55]. MAC-associated interactions involving GAS6-TAM and CX3CL1-CX3CR1 were enriched in both inactive and cystic states, consistent with immune-mediated modulation of ovarian function [56]. The shift toward fewer but stronger dominant signaling axes in cystic ovaries indicates that normal regulatory networks may be replaced by pathological circuits that sustain a non-ovulatory state.

NicheNet analysis further supported state-specific GC regulation. Follicular stage GC programs were driven by ligands including *INHBA*, *AMH*, and *BMP4*, with downstream targets such as *CYP19A1* and *HSD17B1*, consistent with TGF-β-mediated regulation of FSH responsiveness [40,52]. Luteal and pregnancy states showed a shift toward angiogenic and immune-modulatory ligands, including *ANGPT1* and *A2M*, reflecting requirements for vascular stabilization and immune adaptation [57]. In contrast, cystic ovaries were characterized by pro-inflammatory ligands including *TNF*, *IL1B*, and *CCL*/*CXCL* family members, reinforcing the concept that inflammatory dysregulation contributes to cyst pathology [51,54].

The scFEA analysis suggested subtype- and state-associated differences in predicted metabolic flux. Inferred citrate-related patterns in cumulus GCs and succinyl-CoA-related patterns in mural and luteinizing GCs were compatible with the energetic, lipid-metabolic, and steroidogenic requirements previously described for bovine follicular and luteal cells [58,59]. Predicted alterations involving malonyl-CoA, crotonoyl-CoA, and hexenoyl-CoA in atretic GCs suggested possible changes in fatty-acid metabolism, consistent with evidence that altered fatty-acid oxidation and lipid stress can affect bovine cumulus and granulosa-cell function [60,61]. Polyamine metabolism, particularly putrescine conversion to N-acetylputrescine in mural GCs, and differential GABA pathway activity associated with *ABAT* expression, suggest roles in supporting follicular maturation and metabolic resilience [62,63]. Disrupted one-carbon metabolism in atretic GCs, indicated by homocysteine and adenosine accumulation, may further compromise oocyte and follicle quality [64,65]. Nevertheless, scFEA infers metabolic flux from transcript abundance and metabolic-network constraints; it does not directly measure metabolite concentrations, enzyme activity, substrate utilization, or reaction rates [29]. Accordingly, the reported metabolic patterns should be viewed as hypothesis-generating predictions that require validation through targeted metabolomics, enzyme assays, or isotope-tracing experiments.

This study has several limitations that should be acknowledged. First, the study included 18 animals, with only three cows in each of the inactive and luteal cystic groups, limiting the power to characterize the full extent of inter-animal heterogeneity. For hormone analyses, the individual cow was treated as the biological replicate, and technical triplicates were averaged to obtain one value per animal. For snRNA-seq, each animal was processed as an independent sample and animal identity was retained during data integration; however, the limited number of animals per state restricted formal estimation of donor-level variation in several exploratory cell-level analyses. Second, the cross-sectional design means that pseudotime represents an inferred transcriptional ordering rather than actual biological time or direct lineage progression. Third, pooling tissues from different ovarian regions removed spatial information, and the observed cell-type proportions represent relative nuclear composition rather than absolute cell abundance. Fourth, the once-daily gonadotropin measurements were insufficient to characterize pulsatile FSH and LH secretion, and the follicular-fluid measurements could not distinguish systemic transfer from local production. Fifth, the inferred trajectories, co-expression modules, ligand-receptor relationships, ligand-target associations, and metabolic fluxes require spatial, protein-level, metabolic, and functional validation. Finally, follicular cysts were not collected as a separate group, which also limits the pathological spectrum represented in this atlas. Future studies using larger cohorts, animal-level statistical models, spatial profiling, proteomics, metabolomics, and functional perturbation experiments will be required to validate these candidate mechanisms.

## 5. Conclusions

This study presents a multi-state single-nucleus transcriptomic profile of the Holstein cow ovary and describes state-associated variation in ovarian cell composition and granulosa-cell transcriptional programs. Inactive ovaries were associated with lower levels of predicted intercellular communication, whereas luteal cystic ovaries showed relative enrichment of immune- and inflammation-related transcriptional signatures, together with differences in steroidogenic and predicted metabolic features. These computationally inferred patterns are hypothesis-generating and may inform the design of future experimental validation studies.

## Figures and Tables

**Table 1:** Hormone indicators in serum of Holstein cows.

Item	Luteal	Follicular	Pregnancy	Cyst	Inactive	*p*-Value
E_2_, pg/mL	3.48 ± 0.66 ^b^	10.70 ± 1.30 ^c^	7.41 ± 1.02 ^d^	3.03 ± 0.65 ^ab^	1.00 ± 0.51 ^a^	1.78 × 10^−8^
FSH, ng/mL	1.55 ± 0.35 ^bc^	2.32 ± 0.34 ^a^	1.10 ± 0.21 ^c^	1.70 ± 0.27 ^abc^	2.07 ± 0.44 ^ab^	0.00157
LH, ng/mL	0.77 ± 0.31 ^ab^	1.52 ± 0.34 ^ac^	0.62 ± 0.08 ^b^	0.92 ± 0.28 ^ab^	2.14 ± 0.75 ^c^	0.001
P_4_, ng/mL	4.27 ± 0.83 ^c^	0.32 ± 0.11 ^b^	8.69 ± 2.03 ^d^	18.77 ± 3.60 ^a^	0.41 ± 0.22 ^bc^	2.8 × 10^−8^

Values are presented as mean ± SD. Different superscript letters within the same row indicate significant differences between groups (Tukey’s HSD post hoc test, *p* < 0.05).

**Table 2:** Hormone indicators in follicular fluid of Holstein cows.

Item	Luteal	Follicular	Pregnancy	Cyst	*p*-Value
E_2_, ng/mL	15.04 ± 8.29 ^a^	110.17 ± 15.88 ^b^	83.37 ± 37.96 ^b^	7.87 ± 0.6 ^a^	9.946 × 10^−5^
FSH, pg/mL	81.19 ± 3.53 ^ab^	103.46 ± 19.03 ^b^	83.78 ± 4.83 ^ab^	75.19 ± 2.67 ^a^	0.019
LH, ng/mL	2.25 ± 0.50 ^a^	1.94 ± 0.25 ^a^	1.75 ± 0.39 ^a^	2.30 ± 0.11 ^a^	0.182
P_4_, ng/mL	39.89 ± 19.24 ^b^	26.86 ± 10.92 ^b^	81.83 ± 12.15 ^b^	767.20 ± 154.05 ^a^	4.421 × 10^−8^
E_2_/P_4_	0.43 ± 0.20 ^a^	5.33 ± 4.09 ^b^	1.06 ± 0.54 ^ab^	0.010 ± 0.00 ^a^	0.020

Values are presented as mean ± SD. Different superscript letters within the same row indicate significant differences between groups (Tukey’s HSD post-hoc test, *p* < 0.05).

## Data Availability

The raw single-nucleus RNA sequencing data of bovine ovaries generated in this study have been deposited in the National Center for Biotechnology Information (NCBI) under BioProject ID: PRJNA1440217. The dataset is currently under private access and will be made publicly available upon acceptance or publication of this article. For editorial and reviewer evaluation, private access to the dataset is available through the following reviewer link: https://dataview.ncbi.nlm.nih.gov/object/PRJNA1440217?reviewer=216h8v3nqb702slmtel3gm1s11 (accessed on 3 August 2026) Additional processed data, metadata, and materials supporting the findings of this study are available from the corresponding author upon reasonable request.

## Supplementary Materials

The following supporting information can be downloaded at: https://www.mdpi.com/article/10.3390/biology15151327/s1, Table S1. Specifications of the ELISA kits used for hormone measurements. Table S2. Raw sequencing data and quality control of Holstein cows. Table S3. The GO enrichment terms of the differentially expressed cluster genes at the first node of the monocle analysis. Table S4. Intersection analysis between hdWGCNA module genes and Monocle HVGs across five ovarian physiological states. Table S5. The pathway differences among various cell types of the ovary under different physiological conditions in CellChat. Table S6. The results of the scFEA metabolic flux analysis for mural GC and luteinizing GC. Figure S1. Single-cell Transcriptomic Landscape of Bovine Ovaries Across Different Physiological States. A UMAP visualization of integrated snRNA-seq data from five groups: Luteal, Follicular, Inactive, Pregnancy, and Cystic ovaries. Cells are colored by their corresponding Seurat clusters, suggesting the conservation and heterogeneity of cellular populations across groups. B Dot plot showing the expression of signature marker genes used for annotating major cell types across Seurat clusters. C Spearman correlation heatmap between Seurat clusters and five GC subtypes, based on representative marker genes. D Dot plots showing the expression patterns of marker genes across clusters and GC subtypes. E Stacked bar plot showing the relative proportion of GC subtypes in each physiological group. F Cell state distribution of GCs from Inactive, Luteal, Pregnancy, Cyst, and Follicular phase along the developmental component axis. Figure S2. HdWGCNA analysis of GCs in different state and functional analysis of haWGCNA-specific genes. A Dot plot illustrates the average expression and percent of cells expressing genes within each identified co-expression module across major ovarian cell lineages in the luteal phase. B The density plots display the distribution of kME values for each color-coded module in pregnancy stage, indicating the connectivity and assignment strength of genes within their respective modules. C The dendrogram shows the clustering of genes within GCs in the inactive ovary. D GO enrichment analysis of key GC modules of inactive ovary. E Dot plot further validating the cell-type-specific activity of co-expression modules, highlighting modules with high specificity to GCs of cyst ovary. F GO enrichment analysis of hdWGCNA module genes in cyst ovary. Figure S3. Landscape of cell-cell communication in ovaries across different physiological states. A The circle plots visualize the number of interactions between major cell types in follicular, luteal, inactive, pregnancy, and cystic ovaries. B-E Heatmaps showing the relative strength of specific signaling pathways across different cell lineages, comparing the follicular group with luteal, pregnancy, cyst, and cystic ovaries. Figure S4. L-R pairs interaction patterns across physiological states. A-D Dot plots displaying the specific molecular interactions between sender and receiver cell types, comparing the follicular group with luteal, pregnancy, cyst, and cystic ovaries. Figure S5. NicheNet analysis of differential L-R pair activities in follicular phase compared with other physiological states. A, C, E and G The heatmap plots illustrate the prioritized ligands identified in sender cell types that are differentially active in the follicular ovary compared to luteal, pregnancy, inactive, and cystic ovaries. It further displays the corresponding expression changes in predicted target genes in GCs, revealing the downstream transcriptional programs driven by these upstream signals. B, D, F and H Circos plots visualize the links between ligands expressed by various sender cell populations and their corresponding receptors on receiver GCs in the follicular ovary compared to luteal, pregnancy, inactive, and cystic ovaries. The thickness of the connecting lines reflects the prior interaction potential. Figure S6. ScFEA-based profiling of metabolic flux changes across ovarian physiological states and GC subtypes. A The heatmap illustrates the relative activity levels of the top 50 across five states. B The dot plot illustrates the inferred metabolic activity of key metabolites specifically associated with the ovulation process within GC subtypes.

## Abbreviations

4-PL	Four-parameter logistic
ANOVA	Analysis of variance
ANGPT	Angiopoietin signaling pathway
BMP	Bone Morphogenetic Protein signaling pathway
C	Clusters
CALCR	Calcitonin receptor signaling pathway
CCA	Canonical correlation analysis
COD	Cystic ovarian disease
CSF	Colony-stimulating factor signaling pathway
DE	Differential expression
DEGs	Differentially expressed genes
DPBS	Dulbecco’s Phosphate-buffered saline
E_2_	Estradiol
ECM	Extracellular matrix
ELISA	Enzyme-linked immunosorbent assay
FGF	Fibroblast growth factor signaling pathway
FAO	Fatty acid β-oxidation
FSH	Follicle-stimulating hormone
GAS	Growth arrest-specific protein signaling pathway
GALECTIN	Galectin signaling pathway
GCs	Granulosa cells
GEM	Gel bead-in-emulsion
GO	Gene Ontology
hdWGCNA	High-dimensional weighted gene co-expression network analysis
HVGs	Highly variable genes
IGF	Insulin-like growth factor signaling pathway
KITL	KIT ligand signaling pathway
kME	Module eigengene-based connectivity
LH	Luteinizing hormone
L-R	Ligand-receptor
Mac	Macrophages
MNN	Mutual nearest neighbors
NK	Natural killer cells
NRG	Neuregulin signaling pathway
P_4_	Progesterone
PARs	Protease-activated receptors signaling pathway
PCA	Principal component analysis
PCR	Polymerase chain reaction
PI3K-AKT	Phosphatidylinositol 3-kinase-protein kinase B signaling pathway
PTN	Pleiotrophin signaling pathway
RESISTIN	Resistin signaling pathway
scFEA	Single-cell flux estimation analysis
SEMA3	Semaphorin 3 signaling pathway
SMC	Smooth muscle cells
snRNA-seq	Single-nucleus RNA sequencing
STAR	Spliced Transcripts Alignment to a Reference
TCA	Tricarboxylic acid cycle
TGF-β	Transforming growth factor-β signaling pathway
TNF	Tumor necrosis factor signaling pathway
UMAP	Uniform manifold approximation and projection
UMIs	Unique molecular identifiers
VEGF	Vascular endothelial growth factor signaling pathway
VISFATIN	Visfatin signaling pathway
Wnt	Wingless/Integrated signaling pathway
