# Supplementary material for: Single-Nucleus Transcriptomics Reveals Granulosa Cell Heterogeneity and Microenvironmental Remodeling Across Bovine Ovarian States
Source: Biology (Basel). 2026 Aug 6;15(15):1327. doi: 10.3390/biology15151327 (PMC13464624; doi:10.3390/biology15151327)
Supplement: Supplementary file 1 [file biology-15-01327-s001.zip › Figure S1. Single-cell Transcriptomic Landscape of Bovine Ovaries Across Different Physiological States..pdf]

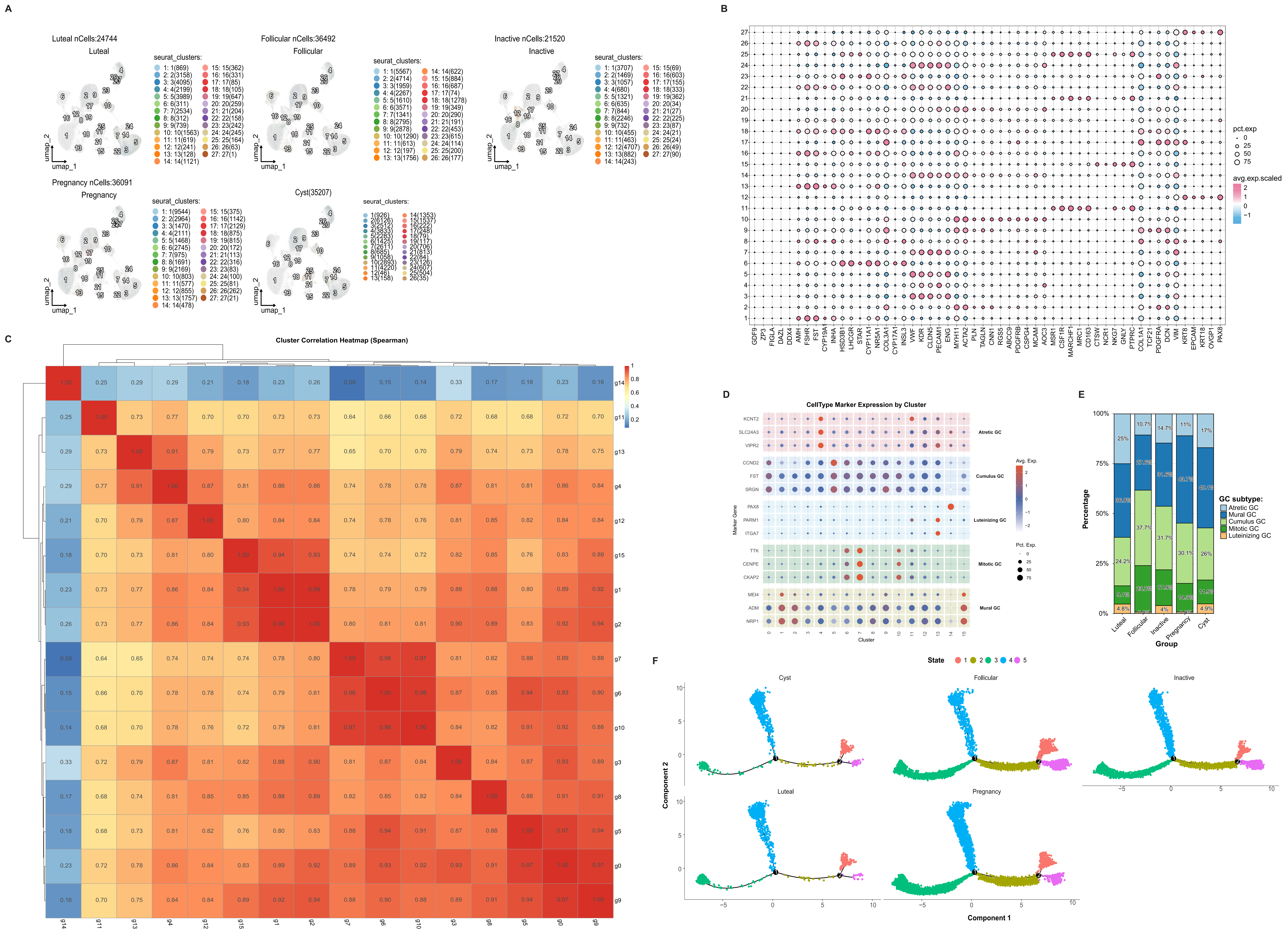
A
Luteal nCells:24744
Luteal
Follicular nCells:36492
Follicular
Inactive nCells:21520
Inactive
Pregnancy nCells:36091
Pregnancy
Cyst(35207)
seurat_clusters:
umap_1
umap_2
B
pct.exp
avg.exp.scaled
C
Cluster Correlation Heatmap (Spearman)
D
CellType Marker Expression by Cluster
Atretic GC
Cumulus GC
Luteinizing GC
Mitotic GC
Mural GC
Marker Gene
Cluster
Avg. Exp.
Pct. Exp.
E
Percentage
Group
GC subtype:
Atretic GC
Mural GC
Cumulus GC
Mitotic GC
Luteinizing GC
Luteal
Follicular
Inactive
Pregnancy
Cyst
F
State
Cyst
Follicular
Inactive
Luteal
Pregnancy
Component 2
Component 1
