# Supplementary material for: Single-Nucleus Transcriptomics Reveals Granulosa Cell Heterogeneity and Microenvironmental Remodeling Across Bovine Ovarian States
Source: Biology (Basel). 2026 Aug 6;15(15):1327. doi: 10.3390/biology15151327 (PMC13464624; doi:10.3390/biology15151327)
Supplement: Supplementary file 1 [file biology-15-01327-s001.zip › Figure S2. HdWGCNA analysis of GCs in different state and functional analysis of haWGC-NA-specific genes. .pdf]

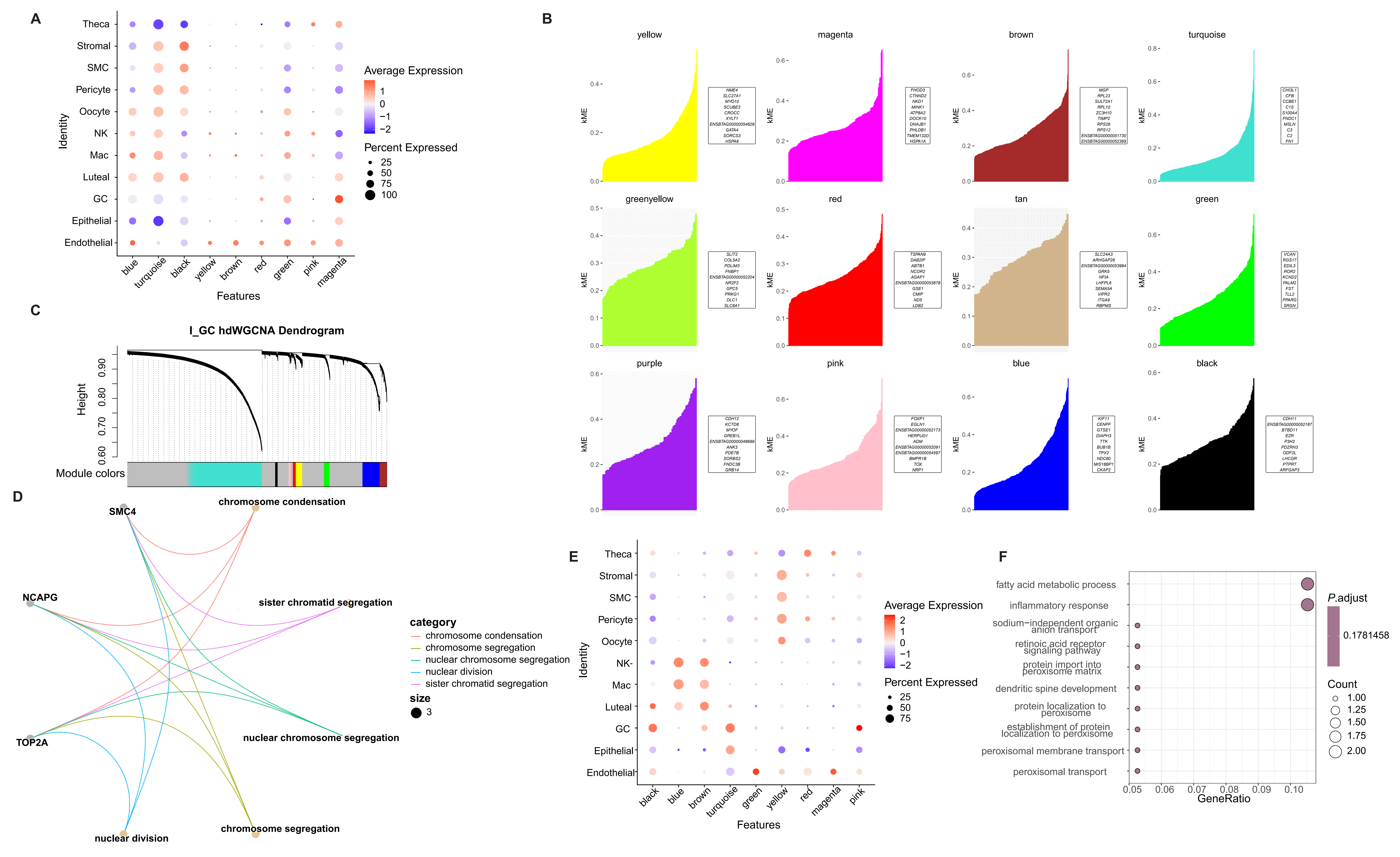
A
Theca
Stromal
SMC
Pericyte
Oocyte
NK
Mac
Luteal
GC
Epithelial
Endothelial
Identity
blue
turquoise
black
yellow
brown
red
green
pink
magenta
Features
Average Expression
1
0
−1
−2
Percent Expressed
25
50
75
100
B
yellow
magenta
brown
turquoise
greenyellow
red
tan
green
purple
pink
blue
black
kME
C
I_GC hdWGCNA Dendrogram
Height
0.60
0.70
0.80
0.90
Module colors
D
SMC4
chromosome condensation
NCAPG
sister chromatid segregation
TOP2A
nuclear chromosome segregation
nuclear division
chromosome segregation
category
chromosome condensation
chromosome segregation
nuclear chromosome segregation
nuclear division
sister chromatid segregation
size
3
E
Theca
Stromal
SMC
Pericyte
Oocyte
NK
Mac
Luteal
GC
Epithelial
Endothelial
Identity
black
blue
brown
turquoise
green
yellow
red
magenta
pink
Features
Average Expression
2
1
0
−1
−2
Percent Expressed
25
50
75
F
fatty acid metabolic process
inflammatory response
sodium−independent organic anion transport
retinoic acid receptor signaling pathway
protein import into peroxisome matrix
dendritic spine development
protein localization to peroxisome
establishment of protein localization to peroxisome
peroxisomal membrane transport
peroxisomal transport
0.05
0.06
0.07
0.08
0.09
0.10
GeneRatio
P.adjust
0.1781458
Count
1.00
1.25
1.50
1.75
2.00
