# Supplementary material for: Single-Nucleus Transcriptomics Reveals Granulosa Cell Heterogeneity and Microenvironmental Remodeling Across Bovine Ovarian States
Source: Biology (Basel). 2026 Aug 6;15(15):1327. doi: 10.3390/biology15151327 (PMC13464624; doi:10.3390/biology15151327)
Supplement: Supplementary file 1 [file biology-15-01327-s001.zip › Figure S3. Landscape of cell-cell communication in ovaries across different physiological states. .pdf]

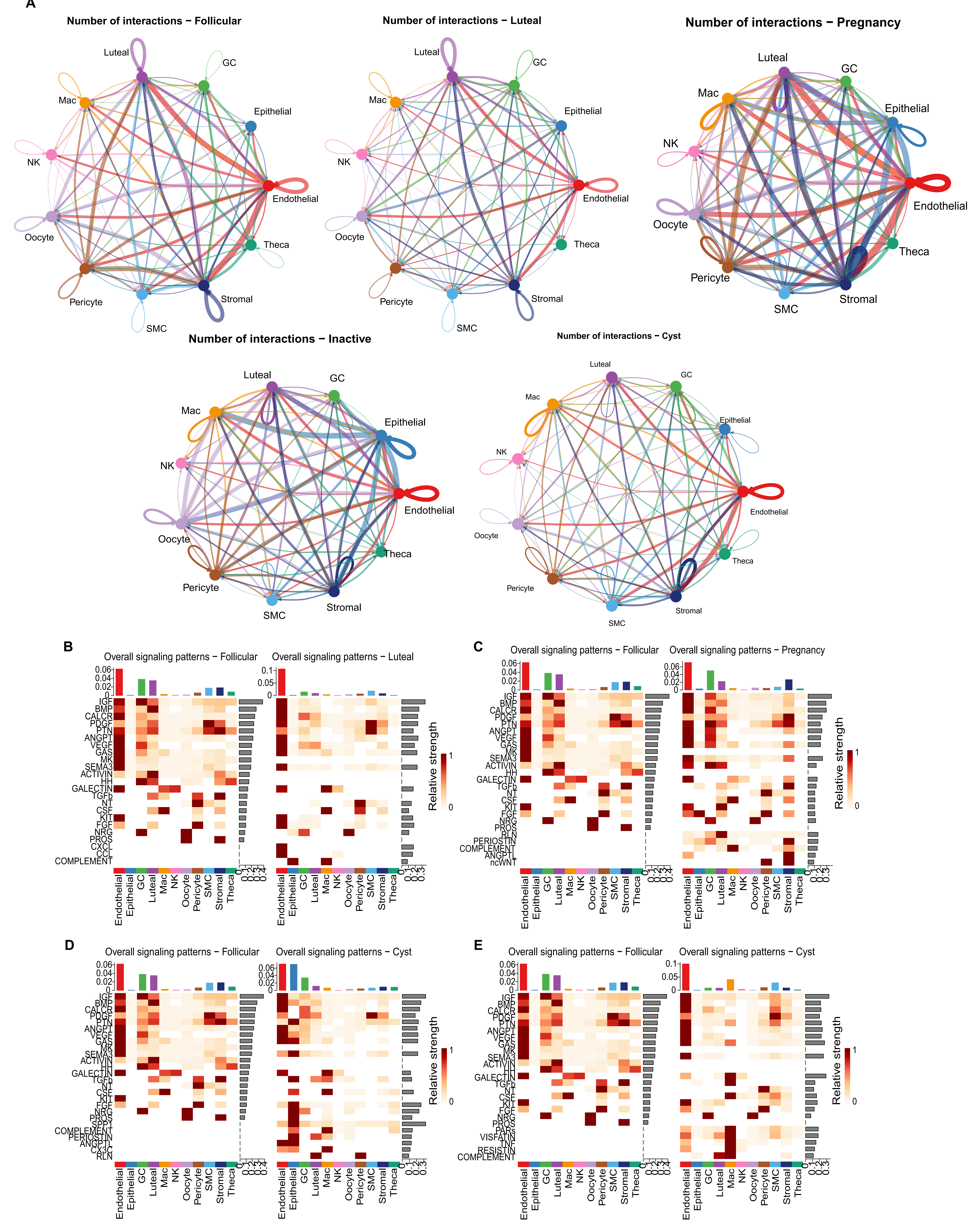
A
Number of interactions − Follicular
Number of interactions − Luteal
Number of interactions − Pregnancy
Number of interactions − Inactive
Number of interactions − Cyst
Luteal
GC
Mac
Epithelial
NK
Endothelial
Oocyte
Theca
Pericyte
SMC
Stromal
B
Overall signaling patterns − Follicular
Overall signaling patterns − Luteal
C
Overall signaling patterns − Follicular
Overall signaling patterns − Pregnancy
D
Overall signaling patterns − Follicular
Overall signaling patterns − Cyst
E
Overall signaling patterns − Follicular
Overall signaling patterns − Cyst
IGF
BMP
CALCR
PDGF
PTN
ANGPT
VEGF
GAS
MK
SEMA3
ACTIVIN
HH
GALECTIN
TGFb
NT
CSF
KIT
FGF
NRG
PROS
CXCL
CCL
COMPLEMENT
RLN
PERIOSTIN
ANGPTL
ncWNT
SPP1
CX3C
PARs
VISFATIN
TNF
RESISTIN
Relative strength
Endothelial
Epithelial
GC
Luteal
Mac
NK
Oocyte
Pericyte
SMC
Stromal
Theca
