# Supplementary material for: Single-Nucleus Transcriptomics Reveals Granulosa Cell Heterogeneity and Microenvironmental Remodeling Across Bovine Ovarian States
Source: Biology (Basel). 2026 Aug 6;15(15):1327. doi: 10.3390/biology15151327 (PMC13464624; doi:10.3390/biology15151327)
Supplement: Supplementary file 1 [file biology-15-01327-s001.zip › Figure S4. L-R pairs interaction patterns across physiological states. .pdf]

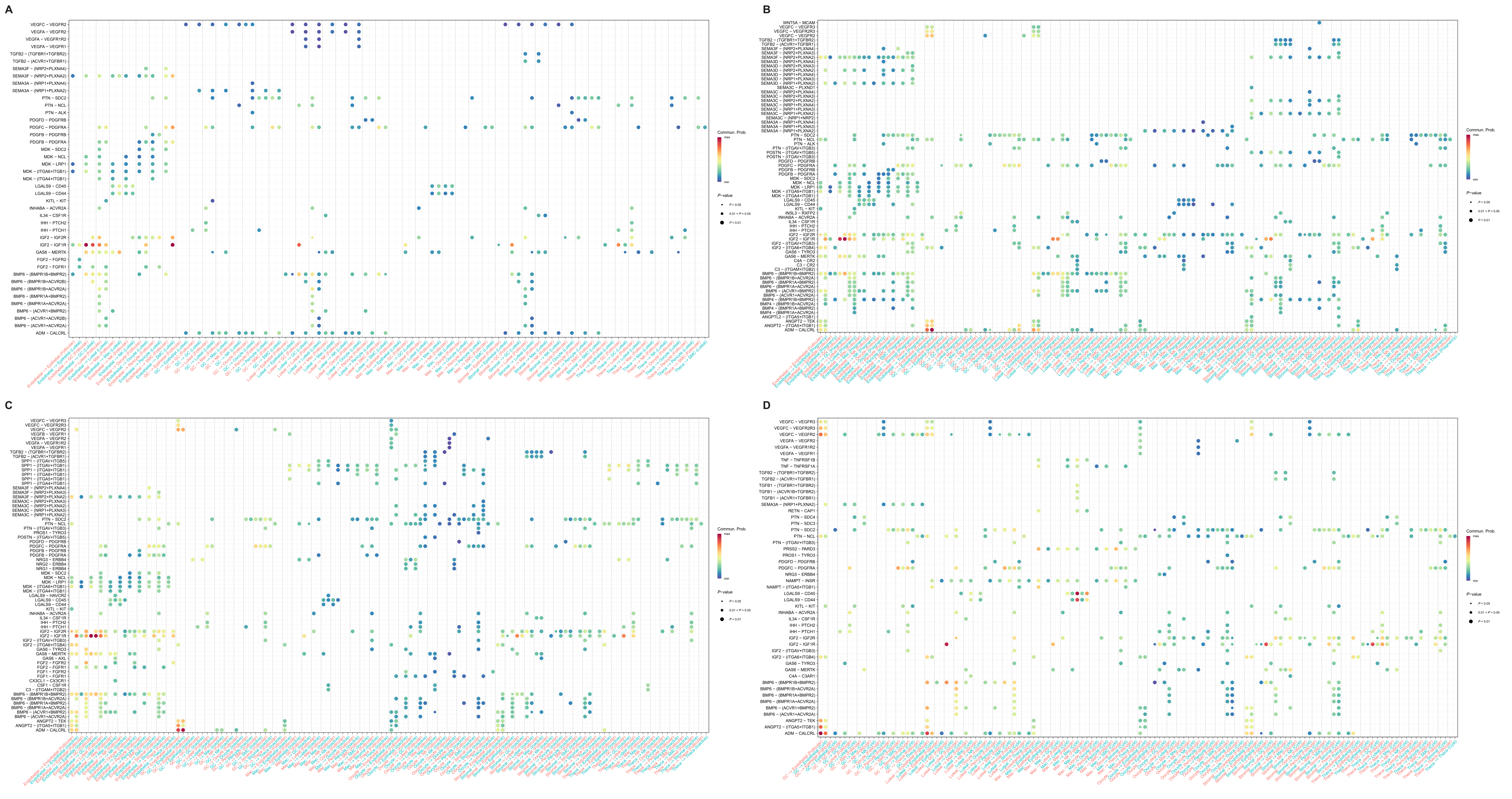
A
B
C
D
Commun. Prob.
max
min
P-value
P > 0.05
0.01 < P < 0.05
P < 0.01
