# Supplementary material for: Single-Nucleus Transcriptomics Reveals Granulosa Cell Heterogeneity and Microenvironmental Remodeling Across Bovine Ovarian States
Source: Biology (Basel). 2026 Aug 6;15(15):1327. doi: 10.3390/biology15151327 (PMC13464624; doi:10.3390/biology15151327)
Supplement: Supplementary file 1 [file biology-15-01327-s001.zip › Figure S5. NicheNet analysis of differential L-R pair activities in follicular phase compared with other physiological states..pdf]

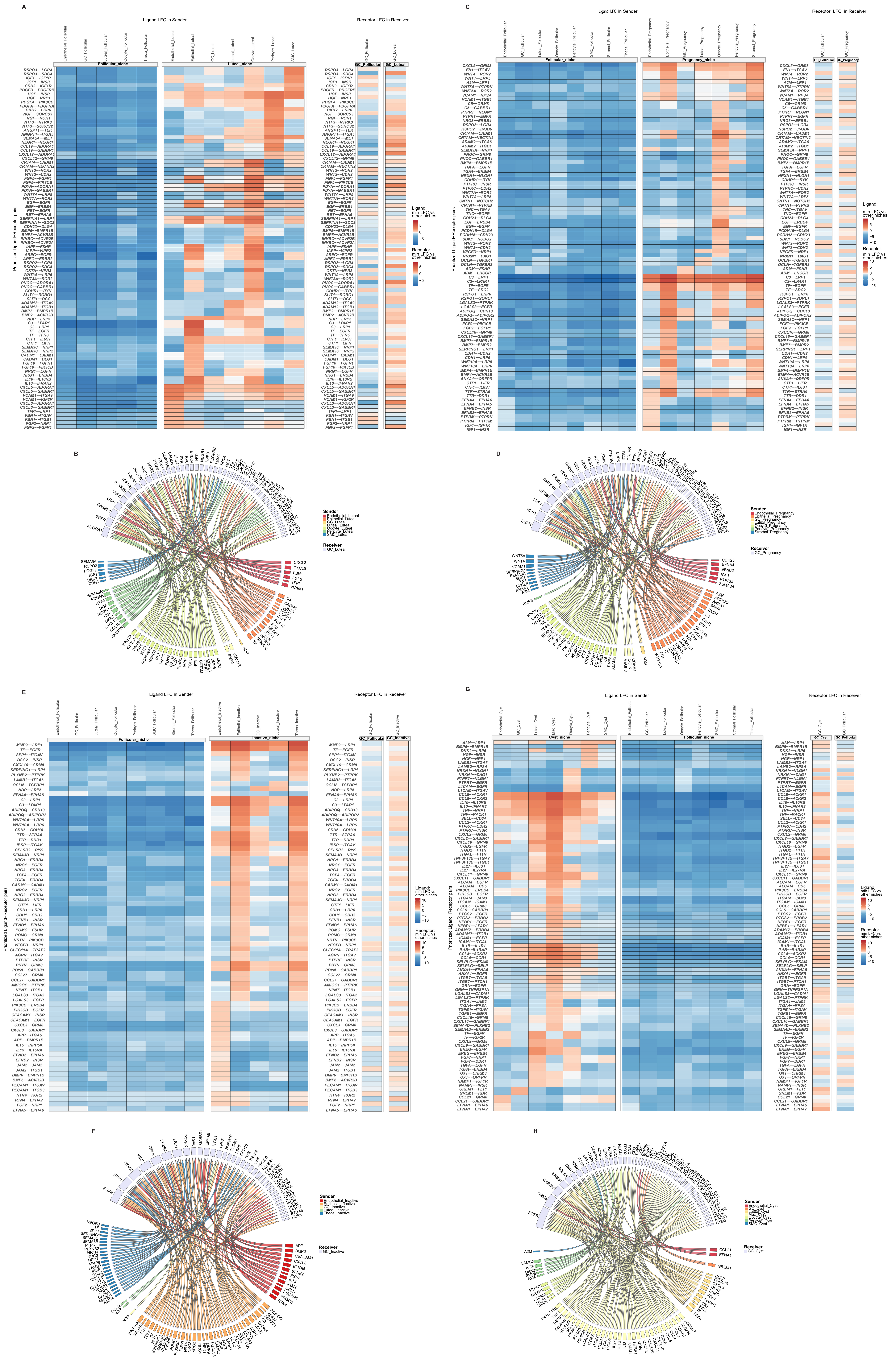

A
Ligand LFC in Sender
Receptor LFC in Receiver
Follicular_niche
Luteal_niche
GC_Follicular
GC_Luteal
Prioritized Ligand–Receptor pairs
Ligand:
min LFC vs
other niches
Receptor:
min LFC vs
other niches
B
Sender
Endothelial_Luteal
Epithelial_Luteal
GC_Luteal
Luteal_Luteal
Oocyte_Luteal
Pericyte_Luteal
SMC_Luteal
Receiver
GC_Luteal
C
Ligand LFC in Sender
Receptor LFC in Receiver
Follicular_niche
Pregnancy_niche
GC_Follicular
GC_Pregnancy
Prioritized Ligand–Receptor pairs
Ligand:
min LFC vs
other niches
Receptor:
min LFC vs
other niches
D
Sender
Endothelial_Pregnancy
Epithelial_Pregnancy
GC_Pregnancy
Luteal_Pregnancy
Oocyte_Pregnancy
Pericyte_Pregnancy
Stromal_Pregnancy
Receiver
GC_Pregnancy
E
Ligand LFC in Sender
Receptor LFC in Receiver
Follicular_niche
Inactive_niche
GC_Follicular
GC_Inactive
Prioritized Ligand–Receptor pairs
Ligand:
min LFC vs
other niches
Receptor:
min LFC vs
other niches
F
Sender
Endothelial_Inactive
Epithelial_Inactive
GC_Inactive
Luteal_Inactive
Theca_Inactive
Receiver
GC_Inactive
G
Ligand LFC in Sender
Receptor LFC in Receiver
Cyst_niche
Follicular_niche
GC_Cyst
GC_Follicular
Prioritized Ligand–Receptor pairs
Ligand:
min LFC vs
other niches
Receptor:
min LFC vs
other niches
H
Sender
Endothelial_Cyst
GC_Cyst
Luteal_Cyst
Mac_Cyst
Oocyte_Cyst
Pericyte_Cyst
SMC_Cyst
Receiver
GC_Cyst
