# Supplementary material for: Single-Nucleus Transcriptomics Reveals Granulosa Cell Heterogeneity and Microenvironmental Remodeling Across Bovine Ovarian States
Source: Biology (Basel). 2026 Aug 6;15(15):1327. doi: 10.3390/biology15151327 (PMC13464624; doi:10.3390/biology15151327)
Supplement: Supplementary file 1 [file biology-15-01327-s001.zip › Figure S6. ScFEA-based profiling of metabolic flux changes across ovarian physiological states and GC subtypes. .pdf]

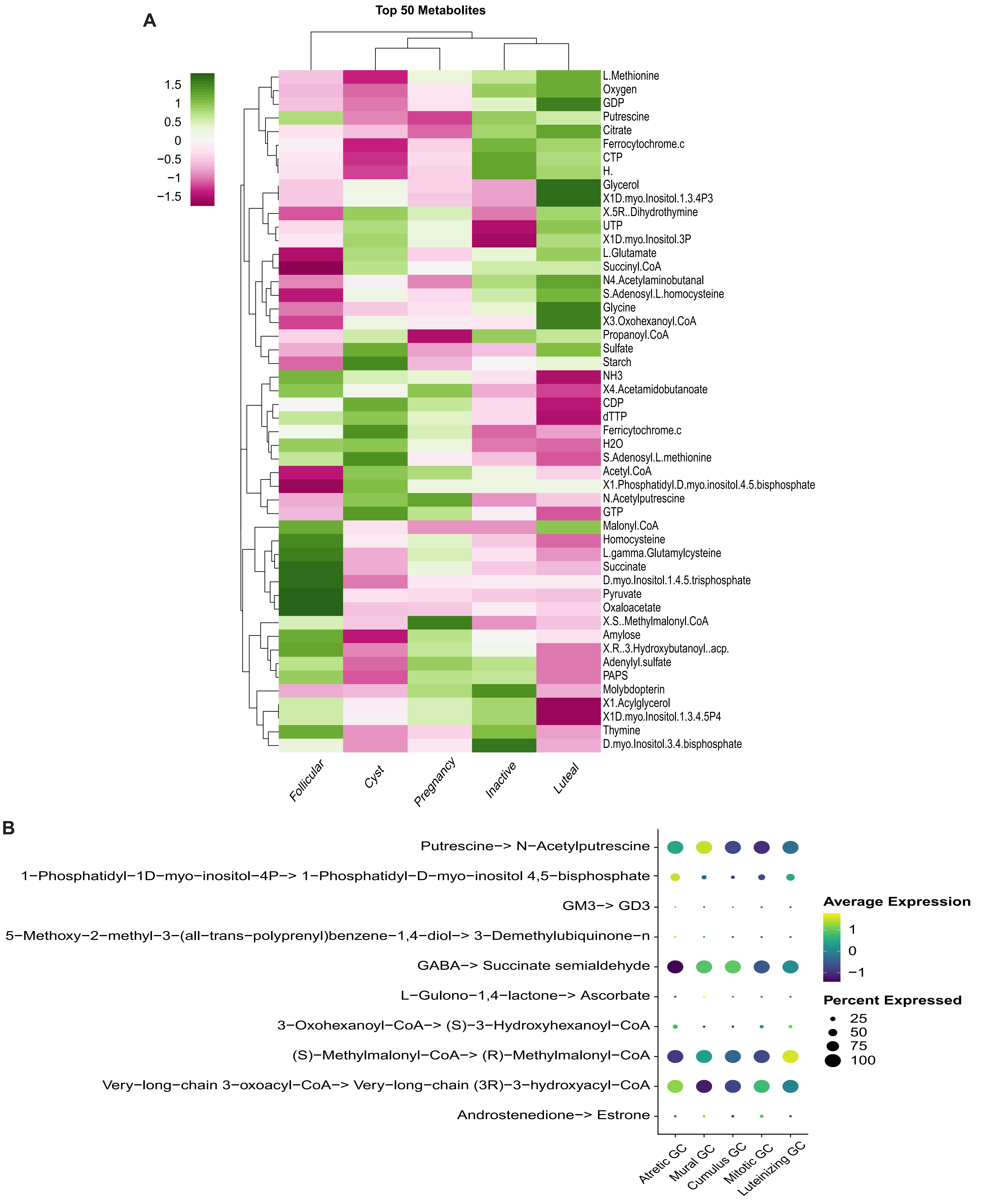
A
Top 50 Metabolites
1.5
1
0.5
0
−0.5
−1
−1.5
L.Methionine
Oxygen
GDP
Putrescine
Citrate
Ferrocytochrome.c
CTP
H.
Glycerol
X1D.myo.Inositol.1.3.4P3
X.5R..Dihydrothymine
UTP
X1D.myo.Inositol.3P
L.Glutamate
Succinyl.CoA
N4.Acetylaminobutanal
S.Adenosyl.L.homocysteine
Glycine
X3.Oxohexanoyl.CoA
Propanoyl.CoA
Sulfate
Starch
NH3
X4.Acetamidobutanoate
CDP
dTTP
Ferricytochrome.c
H2O
S.Adenosyl.L.methionine
Acetyl.CoA
X1.Phosphatidyl.D.myo.inositol.4.5.bisphosphate
N.Acetylputrescine
GTP
Malonyl.CoA
Homocysteine
L.gamma.Glutamylcysteine
Succinate
D.myo.Inositol.1.4.5.trisphosphate
Pyruvate
Oxaloacetate
X.S..Methylmalonyl.CoA
Amylose
X.R..3.Hydroxybutanoyl..acp.
Adenylyl.sulfate
PAPS
Molybdopterin
X1.Acylglycerol
X1D.myo.Inositol.1.3.4.5P4
Thymine
D.myo.Inositol.3.4.bisphosphate
Follicular
Cyst
Pregnancy
Inactive
Luteal
B
Putrescine−> N−Acetylputrescine
1−Phosphatidyl−1D−myo−inositol−4P−> 1−Phosphatidyl−D−myo−inositol 4,5−bisphosphate
GM3−> GD3
5−Methoxy−2−methyl−3−(all−trans−polyprenyl)benzene−1,4−diol−> 3−Demethylubiquinone−n
GABA−> Succinate semialdehyde
L−Gulono−1,4−lactone−> Ascorbate
3−Oxohexanoyl−CoA−> (S)−3−Hydroxyhexanoyl−CoA
(S)−Methylmalonyl−CoA−> (R)−Methylmalonyl−CoA
Very−long−chain 3−oxoacyl−CoA−> Very−long−chain (3R)−3−hydroxyacyl−CoA
Androstenedione−> Estrone
Atretic GC
Mural GC
Cumulus GC
Mitotic GC
Luteinizing GC
Average Expression
1
0
−1
Percent Expressed
25
50
75
100
